# An Evaluation of the Plant Density Estimator the Point-Centred Quarter Method (PCQM) Using Monte Carlo Simulation

**DOI:** 10.1371/journal.pone.0157985

**Published:** 2016-06-23

**Authors:** Md Nabiul Islam Khan, Renske Hijbeek, Uta Berger, Nico Koedam, Uwe Grueters, S. M. Zahirul Islam, Md Asadul Hasan, Farid Dahdouh-Guebas

**Affiliations:** 1 Laboratory of Systems Ecology and Resource Management, Département de Biologie des Organismes, Faculté des Sciences, Université Libre de Bruxelles–ULB, Bruxelles, Belgium; 2 Institute of Forest Growth and Forest Computer Sciences, TU Dresden, Tharandt, Germany; 3 Forestry and Wood Technology Discipline, Khulna University, Khulna, Bangladesh; 4 Biodiversity and Ecology Research Unit, Faculty of Sciences and Bio-engineering Sciences, Vrije Universiteit Brussel–VUB, Brussels, Belgium; 5 Plant Production Systems, Wageningen University and Research Centre, Wageningen, Netherlands; USDA-ARS, UNITED STATES

## Abstract

**Background:**

In the Point-Centred Quarter Method (PCQM), the mean distance of the first nearest plants in each quadrant of a number of random sample points is converted to plant density. It is a quick method for plant density estimation. In recent publications the estimator equations of simple PCQM (PCQM1) and higher order ones (PCQM2 and PCQM3, which uses the distance of the second and third nearest plants, respectively) show discrepancy. This study attempts to review PCQM estimators in order to find the most accurate equation form. We tested the accuracy of different PCQM equations using Monte Carlo Simulations in simulated (having ‘random’, ‘aggregated’ and ‘regular’ spatial patterns) plant populations and empirical ones.

**Principal Findings:**

PCQM requires at least 50 sample points to ensure a desired level of accuracy. PCQM with a *corrected* estimator is more accurate than with a previously *published* estimator. The published PCQM versions (PCQM1, PCQM2 and PCQM3) show significant differences in accuracy of density estimation, i.e. the higher order PCQM provides higher accuracy. However, the *corrected* PCQM versions show no significant differences among them as tested in various spatial patterns except in plant assemblages with a strong repulsion (plant competition). If *N* is number of sample points and *R* is distance, the corrected estimator of PCQM1 is 4(4*N* − 1)/(*π* ∑ *R*^2^) but not 12*N*/(*π* ∑ *R*^2^), of PCQM2 is 4(8*N* − 1)/(*π* ∑ *R*^2^) but not 28*N*/(*π* ∑ *R*^2^) and of PCQM3 is 4(12*N* − 1)/(*π* ∑ *R*^2^) but not 44*N*/(*π* ∑ *R*^2^) as published.

**Significance:**

If the spatial pattern of a plant association is random, PCQM1 with a corrected equation estimator and over 50 sample points would be sufficient to provide accurate density estimation. PCQM using just the nearest tree in each quadrant is therefore sufficient, which facilitates sampling of trees, particularly in areas with just a few hundred trees per hectare. PCQM3 provides the best density estimations for all types of plant assemblages including the repulsion process. Since in practice, the spatial pattern of a plant association remains unknown before starting a vegetation survey, for field applications the use of PCQM3 along with the corrected estimator is recommended. However, for sparse plant populations, where the use of PCQM3 may pose practical limitations, the PCQM2 or PCQM1 would be applied. During application of PCQM in the field, care should be taken to summarize the distance data based on ‘the inverse summation of squared distances’ but not ‘the summation of inverse squared distances’ as erroneously published.

## Introduction

Density estimators are used in a wide variety of fields ranging from plant ecology, forestry and demography studies to medical sciences and astronomy. Density of plant populations is generally defined as the number of plants per unit area, which can be estimated by counting plants in plots with a known area. Instead of using plots, density of plant populations can however also be estimated using plotless methods, e.g. Point-Centred Quarter Method (PCQM) [1, 2] among other plotless methods [3–5]. In PCQM, the distance of plants to random sample points is converted to plant density. To address a number of practical problems that arise in some fields, such as mangroves (multiple-stemmed trees, quadrants where no trees are immediately present) the PCQM+ protocol was proposed [6]. The PCQM serves as a suitable method in vegetation study [7] especially when there is an accessibility issue as commonly observed in mangroves [8–11].

Plotless methods are preferred when plot-based (quadrat) sampling would be difficult or too costly [12, 13]. Plotless methods are faster, less laborious and require less equipment. Comparisons of various plotless methods [3, 4, 14] reveal that they have statistical uncertainty and there is no uniformly best plotless method for all types of spatial patterns in vegetation. Although a new composite *k*-tree estimator has been reported to mitigate the statistical bias [15], this still suffers from implementation issues concerning the spatial pattern of plants. As previous studies exist which compare various plotless methods, this review rather focuses on reviewing the different equations used for PCQM.

In vegetation study, there are many approaches, each having its strengths and weaknesses, making them more or less suitable for achieving a given objective. When difficult field conditions exist which make it challenging to access sites and trees (for example mangroves), using PCQM methods is an excellent option providing speedy sampling while requiring few logistics. PCQM allows estimation of plant densities based on scattered points over a larger geographic area than is possible for quadrat sampling. Its main limitation, however, is its bias or statistical uncertainty like any other plotless methods, which is partly related to the number of sample points. In this study, we thus focus on the optimization of PCQM methods varying in the order of considered trees per quadrant and the estimator equation used. Their performance is related to costs and effort, to data quality, and to statistical accuracy and precision.

The pioneer work on PCQM by Cottam and Curtis [2] was further modified by Pollard [16], which improved the statistical bias with PCQM estimator and later on Beasom and Haucke [17] found this method as the best plotless density estimator. In PCQM, the mean distance of the first nearest plant in each of four quadrants of a random sample point is converted to density. The accuracy of PCQM has also been explored through the second order distance (PCQM2 –distance of second nearest plant in each quadrant is measured) as well as 3rd order distance (PCQM3– distance of third nearest plant in each quadrant is measured). It has been argued that higher order PCQM offers better accuracy of density estimation [3, 4]. Based on the first order PCQM estimator [16] and the concept of the *k*-^th^ nearest plant in a circular distance from sample point described by Pollard [16], higher order PCQM density estimators has also been derived, as reported in Engeman et al. [3] and White et al. [4] where the performance of various plotless density estimators have been compared. However, the estimators for simple PCQM (PCQM1) and the higher order ones (PCQM2 and PCQM3) need to be clarified further because of ambiguity in the equations used for PCQM in recent publications [3, 4, 16, 18, 19].

After Cottam [1] and Cottam and Curtis [2] the density (*ρ*) estimator of PCQM [16] stands as

$$\rho =4\;(4N-1)/\left(\pi \sum_{i=1}^{N}\sum_{j=1}^{4}R_{ij}^{2}\right)$$ (1)

Where *R*_*ij*_ = the distance from the *i*^th^ random point to the closest individual in the *j*^th^ quadrant; *N* is the number of random points used; 4 is the number of equiangular sectors about the random sample point and 4*N* is the number of distances measured. After the work of Pollard [16], Engeman et al. [3] followed by White et al. [4] described the second and third order PCQM density (*ρ*) estimators using the following general formula:

$$\rho =Nk(gk-1)/\left(\pi \sum_{i=1}^{N}\sum_{j=1}^{4}R_{(g)ij}^{2}\right)$$ (2)

Where *k* the number of equiangular sectors (quadrants) about the random sample point (*k* is always 4 for PCQM); *g* the number of individuals located in each quadrant and other notations are same as Eq 1. Solving this general equation (Eq 2) for PCQM1, PCQM2 and PCQM3, Engeman et al. [3] and White et al. [4] came to the following equations:

$$\text{PCQM}1,\;\rho =12N/\left(\pi \sum_{i=1}^{N}\sum_{j=1}^{4}R_{(1)ij}^{2}\right)$$ (3)

$$\text{PCQM}2,\;\rho =28N/\left(\pi \sum_{i=1}^{N}\sum_{j=1}^{4}R_{(2)ij}^{2}\right)$$ (4)

$$\text{PCQM}3,\;\rho =44N/\left(\pi \sum_{i=1}^{N}\sum_{j=1}^{4}R_{(3)ij}^{2}\right)$$ (5)

Since the publication from Engeman et al. (1994), these equations have been widely used [3, 4, 20]. In further sections, we will refer to these three equations as the *published* estimators. For PCQM1, it is obvious that the formula (Eq 3) deviates from the one proposed by Pollard [16], who did not propose any formula for PCQM2 and PCQM3. However, based on appropriate interpretation of PCQM1 in Pollard [16] the PCQM2 and PCQM3 can be expressed by the following general equation:

$$\rho =k(gNk-1)/\left(\pi \sum_{i=1}^{N}\sum_{j=1}^{4}R_{(g)ij}^{2}\right)$$ (6)

**Fig 1:**
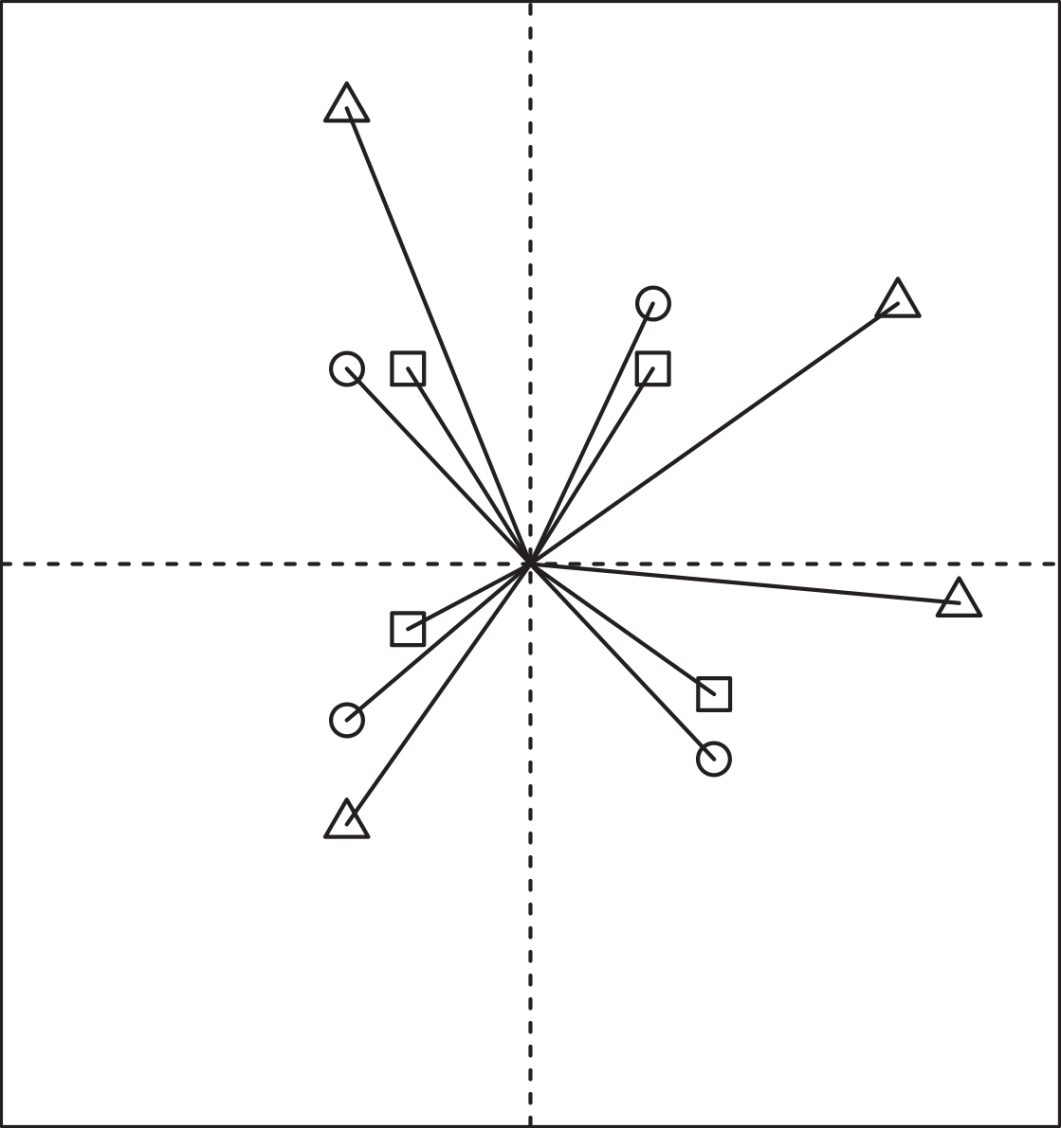
Schematic representation of a PCQM sample point with trees represented as circles, squares or triangles. In this example squares are always the nearest to the sample point and represent trees measured for PCQM, followed by circles for PCQM2 and triangles for PCQM3.

Where *R*_*g*(*ij*)_ is the distance from the *i*^th^ sample point to the *g*^th^ individual in the *j*^th^ quadrant and other notations are same as mentioned above. Solving this general equation for PCQM1, PCQM2 and PCQM3, we come to the following new equations:

$$\text{PCQM}1,\;\rho =4(4N-1)/\left(\pi \sum_{i=1}^{N}\sum_{j=1}^{4}R_{(1)ij}^{2}\right)$$ (7)

$$\text{PCQM}2,\;\rho =4(8N-1)/\left(\pi \sum_{i=1}^{N}\sum_{j=1}^{4}R_{(2)ij}^{2}\right)$$ (8)

$$\text{PCQM}3,\;\rho =4(12N-1)/\left(\pi \sum_{i=1}^{N}\sum_{j=1}^{4}R_{(3)ij}^{2}\right)$$ (9)

where the notations are the same as mentioned above. For more clarity, the terms ‘4’, ‘8’ and ‘12’ in the Eqs 7, 8 and 9 represent the four, eight and 12 plants encountered with the PCQM1, PCQM2 and PCQM3, respectively (Fig 1). In further sections, we will refer to Eqs 7, 8 and 9 as the *corrected* estimators, which is based on appropriate interpretation of the equation for PCQM1 as given byPollard [16]. In our more recent work [21], we have used these equations without any detailed description on PCQM formulae. Comparing the effects of the different formulas on accuracy of PCQM is the focus of this study. As expressed in the Eq 7, PCQM1 stands the same as Pollard [16], which differs with published equation (Eq 3). However, the formulae for PCQM1, PCQM2 and PCQM3 (Eqs 7, 8 and 9) differs from *published* estimators (Eqs 3, 4 and 5) depending on the number of random sample points *N* and the multiplying constants used. For example, when *N* = 10, the numerator in the equations of PCQM1, PCQM2 and PCQM3 stands for 120, 280 and 440, respectively in *published* estimators, i.e. Eqs 3, 4 and 5 but for 156, 316 and 476, respectively in *corrected* estimators, i.e. Eqs 7, 8 and 9. The original concept of PCQM suggests that at least 30 random sample points are required to obtain acceptable results in density estimation through PCQM [2]. In recent publications PCQM1, PCQM2 and PCQM3 have been applied using some constants (12 for PCQM1, 28 for PCQM2 and 44 for PCQM3) in the equations (Eqs 3, 4 and 5). However, in our judgment, there must be 4 objects falling in the imaginary circle of PCQM1, 8 objects in PCQM2 and 12 objects in PCQM3. In the corrected versions of the PCQM equations, we kept these numbers 4, 8 and 12 in the equations for PCQM1, PCQM2 and PCQM3, respectively instead of using those constants (12, 28 and 44). Therefore, in this study, we explore the performance of the *corrected* and *published* estimators for PCQM1, PCQM2 and PCQM3 in plant density estimation. For this purpose, we use some simulated and empirical datasets of plant positions. We hypothesize that the *corrected* estimators are more robust than the *published* estimators and that the higher order PCQM (PCQM2 and PCQM3) shows higher accuracy in the density prediction over first order PCQM.

## Methods

### Ethics Statement

For this study, fieldwork was conducted in a tropical semi-evergreen forest in Lawachara National Park, Moulabi Bazar District, Bangladesh (24°30' N and 091°37' E). A research and field work clearance permit was obtained from the Divisional Forest Officer, Wildlife & Nature Conservation Division, Moulabi Bazar under the Ministry of Environment and Forest, Bangladesh. Another set of data was obtained in Manko wetlands mangrove forest in Okinawa, Japan. Permission was obtained from Manko Waterbird and Wetland Center, Tomigusuku 982, Tomigusuku City, Okinawa prefecture, Japan, under the Ministry of Environment, Japan, which was partially supported by the Ministry of Education, Culture, Sports, Science and Technology, Japan (nos. 16201009 and 16651009).

### Experimental Design

In order to investigate the accuracy of PCQM density estimators on plant populations, simulation experiments were performed using the individual-based modelling platform NetLogo [22]. We developed appropriate codes (S1 Text) to perform experiments using the PCQM with simulated and imported real datasets on plant populations (S1 Fig). Virtual plant assemblages having ‘random’, ‘aggregated’ and ‘regular’ patterns were created (Table 1) through simulation using NetLogo [22] and the ‘spatstat’ package [23] of R-Software version 3.2.2 [24]. Random patterns were created according to the required tree density within the designated area (Table 1). For creating aggregated patterns, both the average radius of the clusters and the aggregation intensity (proportion of population that appears in clusters) were taken into account. Regular patterns were created using different repulsion distances (minimum distance among the neighbours) (Table 1). All the empirical and simulated datasets were assumed to have spatial isotropy and were not assumed to have spatial homogeneity. The aggregation index (*R*) of Clark and Evans [25] was used to express the spatial patterns of data sets used in a quantitative manner (Table 1).

**Table 1:** Characteristics of simulated and empirical datasets having different spatial patterns.

Site	Description	Plot dimension	True density (ha^–1^)	R*
	Natural populations:			
Site 1	12-year old *Kandelia obovata* mangrove stand	20 × 20 m^2^	15,450	0.97
Site 2	20 -year old *K*. *obovata* mangrove stand	20 × 20 m^2^	9,650	1.09
Site 3	Tropical semi-evergreen forest (trees > 5 cm D_130_)	100 × 100 m^2^	795	1.08
	Simulated plant populations:			
1	Random	100 × 100 m^2^	2,000	1.01
2	Random	100 × 100 m^2^	5,000	1.00
3	Random	100 × 100 m^2^	10,000	0.99
4	Aggregated (radius^1^ = 1 m; intensity^2^ = 10%)	100 × 100 m^2^	3,000	0.95
6	Aggregated (radius^1^ = 1 m; intensity^2^ = 30%)	100 × 100 m^2^	3,000	0.87
8	Aggregated (radius^1^ = 1 m; intensity^2^ = 50%)	100 × 100 m^2^	3,000	0.79
9	Aggregated (radius^1^ = 3 m; intensity^2^ = 10%)	100 × 100 m^2^	3,000	0.99
11	Aggregated (radius^1^ = 3 m; intensity^2^ = 30%)	100 × 100 m^2^	3,000	0.94
13	Aggregated (radius^1^ = 3 m; intensity^2^ = 50%)	100 × 100 m^2^	3,000	0.89
14	Regular (repulsion distance^3^ = 0.25 m)	100 × 100 m^2^	3,000	1.03
15	Regular (repulsion distance^3^ = 0.50 m)	100 × 100 m^2^	3,000	1.12
16	Regular (repulsion distance^3^ = 0.75 m)	100 × 100 m^2^	3,000	1.26
17	Regular (repulsion distance^3^ = 1.00 m)	100 × 100 m^2^	3,000	1.40

*Aggregation index (*R*) of Clark and Evans [25] (*R*>1 suggests regularity, *R*<1 suggests aggregation and *R* = 1 suggests randomness)

^1^aggregation radius, i.e., cluster radius

^2^aggregation intensity, i.e., proportion of population that appears in clusters

^3^minimum distance among the neighbours

Empirical datasets of individual tree x-y positions in field plots of 20 m × 20 m from a monospecific mangrove stand (*Kandelia obovata* Sheue, Liu and Yong) in Okinawa, Japan (Table 1) and of 100 m × 100 m in atropical semi-evergreen forest (trees > 5 cm D_130_, diameter at 130 cm of tree height) in Lawachara National Park, Maulvibazar District, Bangladesh (24°30' N and 091°37' E) were used in this study. The young *K*. *obovata* stand showed a semi-aggregated pattern and the old stand showed a semi-regular pattern (Table 1) as tested with pair correlation function of point pattern analysis [23] and the *R* index [25]. The tropical semi-evergreen forest showed a semi-regular pattern (Table 1).These datasets were imported into the NetLogo environment where trees are located identically to the real plot keeping the x-y positions.

Following the ‘virtual ecologist approach’ [26, 27], we applied virtual PCQM sampling to both empirical and simulated datasets in order to estimate the performance of the corrected PCQM estimators. For this, random PCQM sample points (10, 15, 20, 25, 30, 50 and 100 points per simulation) were generated inside the surveyed area excluding a boundary strip of 10% of the length and width of the area to remove the bias associated with edge effects. Then four quadrants were created at each sample point and the distance from the sample points to the desired nearest individuals (Fig 1) in each of four quadrants (depending on the PCQM order) were measured. The distance data were converted into an estimated density in relation to the “true” density related to either the empirical data set or the virtual assemblage simulated by the model as described above. In this paper, we compared the density estimated by the PCQM method against the density of the whole plot. We called the latter “true density” (Table 1) since it represents the one which has to be estimated by the sampling and the PCQM. A total of 1,000 simulations were performed for each sample size and each population.

A detailed description of the model following the ODD (Overview, Design concepts, Details) protocol for describing individual based models [28–30] is provided in Table 2. NetLogo model codes are provided as supplementary information (S1 Text).

**Table 2:** Model description following the ODD protocol [28–30].

**Overview**	
Purpose of the model	The purpose of this study was to revise the plotless density estimator Point-Centred Quarter Method (PCQM) based on simulated as well as empirical datasets in order to observe the accuracy of prediction in first-, second- and third-order PCQM.
State variables and scales	Individuals in the population are described primarily by their position (x-y coordinates).Plot sizes of the simulation area of 100 m × 100 m were used for this study. In each run populations of varying densities ranging from 2,000 to 15,000 individuals ha^–1^. Random PCQM sample points (15, 20, 25, 30, 50 and 100 points per simulation) were generated inside the simulation area. A total of 1,000 simulations were performed for each sample size and each population.
Process overview and scheduling	The following processes occurs each run: establishment of individuals, establishing a random PCQM sample point inside the NetLogo world, creating four quadrants with the sample point in the center, measuring the distance from the sample point to the desired nearest individual (depending on the PCQM order) in each of the four quadrants.
**Design concepts**	
*Emergence*	Individuals emerge randomly, i.e., the spatial distribution of trees is completely random. There is no growth, mortality or any kind of dynamics in the population.
*Interactions*	There is no interaction among the individuals in the population.
*Sensing*	Individuals “sense” the distance of their neighbours.
*Stochasticity*	Individuals establish randomly irrespective of any conditions. PCQM points are obtained randomly but excluding a boundary strip of 10% of the length and width of the NetLogo world to remove the bias resulting from edge effects.
*Observations*	The model provides tracking of all state variables and derives parameters for all individuals.
**Details**	
Initialization	The general settings of the simulation experiments are: (i) The NetLogo world to be initialized by simulated datasets of tree positions with varying densities based on x-coordinates and y-coordinates depending on spatial patterns; (ii) The NetLogo world to be initialized by empirical datasets of trees located identically to the real field plot keeping the original x-y positions of trees (Table 1).
Input	There is no input in this model.
Submodels	
*Description of a single tree*	A tree is described by its x-y position only.
*Tree density*	The model uses published and corrected PCQM estimators of density described in the section of Materials and Methods of this paper.

### Statistical analysis of the results

The relative root mean square error (RRMSE) was used as the basis of comparisons between the different density estimators, where *I* is the number of simulations (1000), $\hat{\rho }$ is the estimated density and *ρ* is the true density in the population, such that:

$$RRMSE=\sqrt{\frac{\sum {\left(\hat{\rho }-\rho \right)}^{2}}{I∙\rho^{2}}}$$ (10)

Along with the RRMSE, in order to detect the bias of the estimated density relative to the true density, the relative bias (RBIAS) was used, where *I*, $\hat{\rho }$ and *ρ* represent the same as Eq 14, such that:

$$RBIAS=\frac{(\sum \hat{\rho }/I)-\rho }{\rho }$$ (11)

In addition, the Wilcoxon rank-sum test (non-parametric equivalent to the Mann-Whitney U test) was used to estimate the significance of differences between the *corrected* and *published* estimators [3, 4] of PCQM. To explore significance of differences among the orders of PCQM estimators (PCQM1, PCQM2 and PCQM3), a non-parametric one-way analysis of variance, the Kruskal-Wallis test, was performed. For multiple comparisons among PCQM versions, a post-hoc analysis [31] with Kruskal-Wallis tests were used. All statistical analyses were performed using R-Software version 3.2.2[24].

## Results

**Fig 2:**
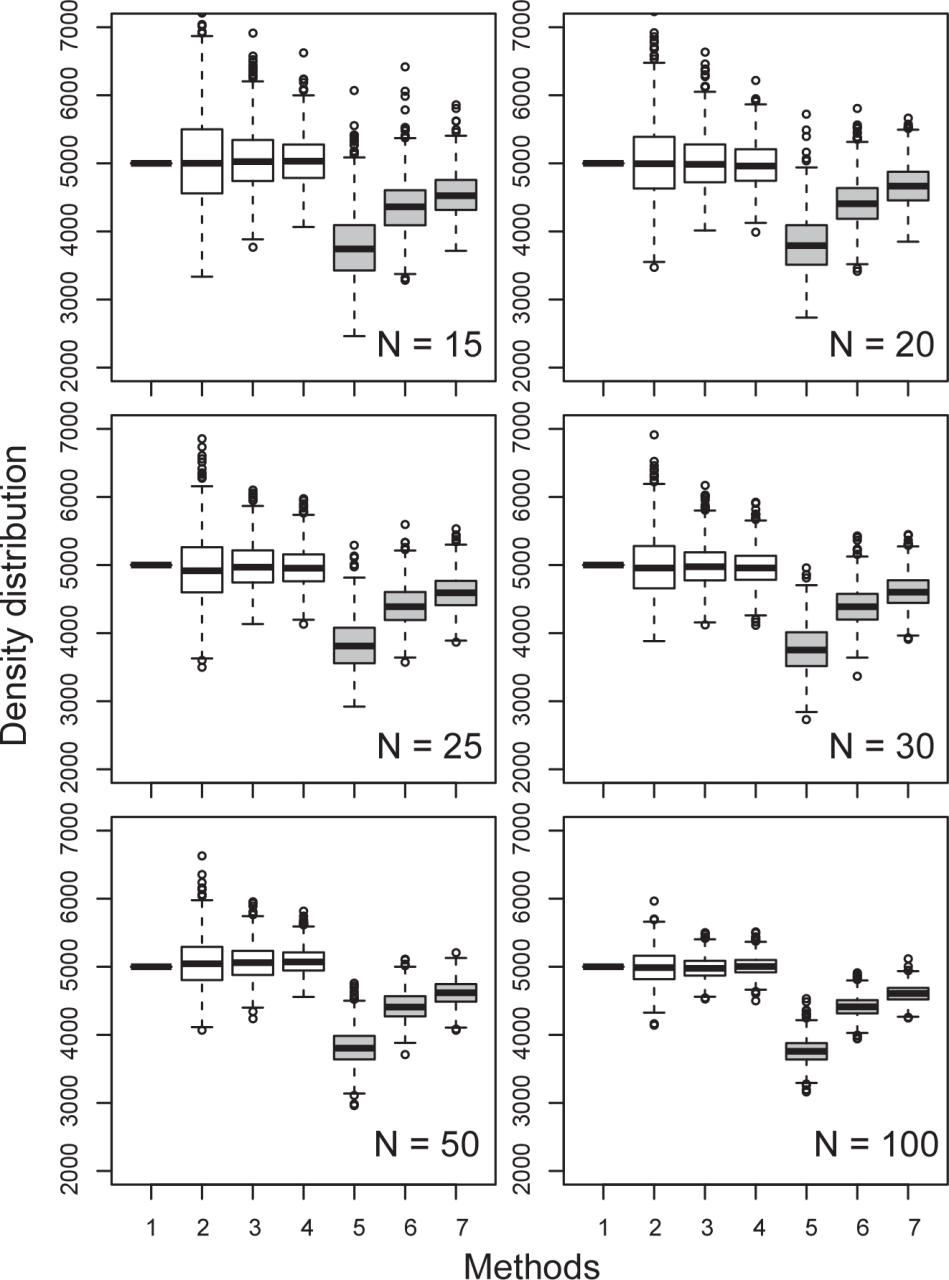
Box plot of the density (individuals ha^–1^) distribution of 1,000 simulations estimated with different methods and varying sample points (N) in a simulated population having a random spatial pattern with a density of 5000 individuals ha^–1^. Boxes with white background represent densities based on *corrected* estimators and those with grey background represent densities based on *published* estimators. Methods: 1 = true density, 2 & 5 = PCQM1, 3 & 6 = PCQM2, 4 & 7 = PCQM3.

Comparison of estimated density based on *corrected* and *published* estimators confirms conspicuous differences in density estimations of plant populations having a wide range of spatial patterns and sample sizes. In plant populations having a ‘random’ spatial pattern, the median values in the estimated densities by *corrected* estimators appear to be very close to the true density even for sample points as few as 15 (Fig 2). The estimated density distribution suggests no apparent differences in the median values among PCQM1, PCQM2 and PCQM3 when the *corrected* estimators are applied (Fig 2). However, using the *published* estimators, the median values of estimated densities are much lower than the true density, and PCQM3 always provides a better prediction of density than PCQM2 followed by PCQM1 (Fig 2). If the *corrected* estimators are applied, it appears that the root mean square error (RRMSE) is the highest when the sample size is very low (10 in this case), and gradually decreases with increasing sample size up to 25, after which it does not show any significant decline when adding random points up to 100 (Table 3). In contrast, the RRMSE values using the *published* PCQM versions are less sensitive to sample size (Table 3). The relative bias (RBIAS) values using the *corrected* PCQM versions (PCQM1, PCQM2 and PCQM3) are very close to zero and again show no differences among the different orders of *corrected* PCQM, while using the *published* PCQM versions the RBIAS values become negative and the higher the order of PCQM, the closer the RBIAS values are to zero. The negative RBIAS in the *published* PCQM versions suggests underestimation of true density.

**Table 3:** The relative root mean square error (RRMSE) and relative bias (RBIAS) with varying true density and “random” spatial pattern.

True density (ha^–1^)	PCQM type		RRMSE						RBIAS					
			Sample points						Sample points					
			15	20	25	30	50	100	15	20	25	30	50	100
2000	*Corrected*	PCQM1	0.131	0.115	0.105	0.090	0.073	0.062	-0.032	0.004	-0.031	-0.011	0.013	0.034
		PCQM2	0.091	0.080	0.080	0.067	0.052	0.051	-0.026	-0.020	-0.027	-0.007	0.016	0.033
		PCQM3	0.073	0.067	0.065	0.055	0.042	0.051	-0.012	-0.020	-0.021	-0.006	0.017	0.040
	*Published*	PCQM1	0.241	0.255	0.216	0.251	0.247	0.244	-0.220	-0.240	-0.201	-0.241	-0.241	-0.241
		PCQM2	0.132	0.140	0.098	0.133	0.117	0.120	-0.106	-0.121	-0.076	-0.121	-0.109	-0.117
		PCQM3	0.097	0.104	0.063	0.091	0.070	0.087	-0.070	-0.086	-0.036	-0.076	-0.060	-0.083
5000	*Corrected*	PCQM1	0.129	0.118	0.100	0.097	0.075	0.052	-0.002	0.009	0.005	-0.014	0.026	0.011
		PCQM2	0.094	0.085	0.068	0.063	0.053	0.035	0.002	-0.008	0.005	-0.006	0.020	0.009
		PCQM3	0.071	0.066	0.058	0.051	0.044	0.030	0.008	-0.004	0.004	-0.006	0.014	0.008
	*Published*	PCQM1	0.248	0.252	0.256	0.238	0.267	0.251	-0.222	-0.237	-0.244	-0.228	-0.262	-0.248
		PCQM2	0.143	0.137	0.135	0.125	0.145	0.129	-0.115	-0.118	-0.120	-0.111	-0.137	-0.125
		PCQM3	0.101	0.098	0.096	0.083	0.096	0.089	-0.073	-0.077	-0.080	-0.069	-0.087	-0.085
10000	*Corrected*	PCQM1	0.128	0.115	0.098	0.091	0.071	0.052	-0.001	-0.013	-0.009	0.011	-0.001	-0.010
		PCQM2	0.095	0.083	0.069	0.063	0.054	0.037	0.003	-0.008	-0.007	0.003	-0.007	-0.004
		PCQM3	0.073	0.066	0.059	0.051	0.043	0.030	0.006	-0.008	-0.010	0.001	-0.009	-0.002
	*Published*	PCQM1	0.260	0.254	0.249	0.259	0.262	0.256	-0.241	-0.241	-0.237	-0.250	-0.256	-0.253
		PCQM2	0.139	0.143	0.129	0.134	0.140	0.133	-0.115	-0.126	-0.114	-0.120	-0.133	-0.129
		PCQM3	0.109	0.102	0.090	0.097	0.097	0.089	-0.083	-0.083	-0.072	-0.083	-0.090	-0.084
15000	*Corrected*	PCQM1	0.126	0.114	0.099	0.090	0.072	0.050	0.012	-0.003	-0.014	-0.004	-0.003	-0.003
		PCQM2	0.092	0.078	0.071	0.065	0.052	0.035	0.014	0.007	-0.007	-0.009	0.003	-0.003
		PCQM3	0.073	0.064	0.061	0.054	0.040	0.028	0.010	0.007	-0.013	-0.008	0.008	0.000
	*Published*	PCQM1	0.252	0.249	0.256	0.250	0.247	0.242	-0.230	-0.234	-0.243	-0.240	-0.241	-0.239
		PCQM2	0.137	0.133	0.145	0.131	0.129	0.121	-0.111	-0.111	-0.131	-0.117	-0.122	-0.117
		PCQM3	0.098	0.096	0.102	0.092	0.093	0.082	-0.072	-0.074	-0.086	-0.077	-0.085	-0.077

In plant populations with “aggregated” spatial pattern the RRMSE and RBIAS values again show no differences among the *corrected* PCQM orders (Table 4). However, the RBIAS values with all the *corrected* PCQM versions become more negative with increasing aggregation intensity (Table 4), but still the *corrected* estimators consistently provide better RBIAS values (closer to zero) than the *published* estimators for any particular aggregation intensity and aggregation radius.

**Table 4:** The relative root mean square error (RRMSE) and relative bias (RBIAS) with “aggregated” spatial pattern, varying aggregation radius (AR), varying aggregation intensity (AI) and a fixed true density of 3000 ha^-1^.

AR (m)	AI (%)	PCQM type		RRMSE						RBIAS					
				Sample points						Sample points					
				15	20	25	30	50	100	15	20	25	30	50	100
1	10	*Corrected*	PCQM1	0.14	0.14	0.11	0.10	0.11	0.08	-0.06	-0.09	-0.06	-0.05	-0.09	-0.06
			PCQM2	0.10	0.11	0.08	0.08	0.09	0.06	-0.06	-0.07	-0.05	-0.05	-0.07	-0.05
			PCQM3	0.08	0.10	0.07	0.07	0.08	0.06	-0.05	-0.07	-0.04	-0.04	-0.07	-0.05
		*Published*	PCQM1	0.30	0.32	0.33	0.31	0.31	0.34	-0.28	-0.31	-0.32	-0.30	-0.30	-0.34
			PCQM2	0.18	0.19	0.20	0.18	0.19	0.22	-0.16	-0.17	-0.19	-0.18	-0.18	-0.21
			PCQM3	0.14	0.14	0.16	0.14	0.14	0.18	-0.13	-0.13	-0.15	-0.13	-0.14	-0.17
1	30	*Corrected*	PCQM1	0.25	0.24	0.23	0.23	0.22	0.25	-0.23	-0.22	-0.22	-0.21	-0.21	-0.24
			PCQM2	0.22	0.22	0.21	0.18	0.19	0.22	-0.20	-0.21	-0.21	-0.17	-0.19	-0.21
			PCQM3	0.20	0.20	0.20	0.16	0.17	0.20	-0.18	-0.19	-0.19	-0.15	-0.17	-0.20
		*Published*	PCQM1	0.39	0.42	0.42	0.40	0.41	0.43	-0.38	-0.42	-0.42	-0.40	-0.41	-0.42
			PCQM2	0.30	0.32	0.30	0.28	0.30	0.32	-0.29	-0.32	-0.30	-0.28	-0.29	-0.32
			PCQM3	0.24	0.27	0.26	0.23	0.25	0.27	-0.23	-0.27	-0.25	-0.23	-0.25	-0.27
1	50	*Corrected*	PCQM1	0.40	0.38	0.39	0.42	0.38	0.38	-0.39	-0.38	-0.39	-0.41	-0.38	-0.38
			PCQM2	0.35	0.34	0.35	0.39	0.35	0.34	-0.34	-0.33	-0.35	-0.38	-0.35	-0.34
			PCQM3	0.31	0.30	0.33	0.35	0.33	0.31	-0.31	-0.29	-0.32	-0.35	-0.32	-0.30
		*Published*	PCQM1	0.54	0.54	0.53	0.51	0.53	0.53	-0.53	-0.53	-0.53	-0.51	-0.53	-0.53
			PCQM2	0.43	0.43	0.42	0.40	0.42	0.42	-0.42	-0.42	-0.42	-0.40	-0.42	-0.42
			PCQM3	0.37	0.36	0.37	0.35	0.37	0.36	-0.37	-0.36	-0.37	-0.35	-0.37	-0.36
3	10	*Corrected*	PCQM1	0.14	0.13	0.10	0.12	0.08	0.07	0.00	-0.07	-0.02	-0.07	-0.05	-0.05
			PCQM2	0.10	0.10	0.08	0.09	0.07	0.05	-0.01	-0.06	-0.02	-0.06	-0.05	-0.04
			PCQM3	0.08	0.08	0.06	0.08	0.06	0.05	-0.01	-0.05	-0.02	-0.05	-0.04	-0.03
		*Published*	PCQM1	0.28	0.27	0.28	0.27	0.27	0.28	-0.26	-0.26	-0.27	-0.27	-0.26	-0.28
			PCQM2	0.17	0.15	0.15	0.17	0.15	0.16	-0.15	-0.14	-0.14	-0.16	-0.14	-0.15
			PCQM3	0.14	0.12	0.11	0.13	0.10	0.11	-0.12	-0.10	-0.09	-0.12	-0.09	-0.11
3	30	*Corrected*	PCQM1	0.16	0.20	0.15	0.18	0.14	0.17	-0.09	-0.16	-0.11	-0.15	-0.13	-0.16
			PCQM2	0.14	0.17	0.13	0.15	0.13	0.13	-0.10	-0.14	-0.11	-0.13	-0.12	-0.13
			PCQM3	0.13	0.15	0.12	0.13	0.12	0.11	-0.10	-0.13	-0.10	-0.12	-0.11	-0.11
		*Published*	PCQM1	0.30	0.35	0.35	0.35	0.35	0.37	-0.28	-0.34	-0.35	-0.34	-0.35	-0.37
			PCQM2	0.19	0.23	0.24	0.23	0.25	0.26	-0.17	-0.22	-0.23	-0.22	-0.25	-0.26
			PCQM3	0.15	0.19	0.20	0.18	0.21	0.21	-0.12	-0.17	-0.19	-0.17	-0.21	-0.21
3	50	*Corrected*	PCQM1	0.30	0.24	0.22	0.24	0.25	0.28	-0.26	-0.21	-0.19	-0.23	-0.24	-0.27
			PCQM2	0.24	0.21	0.19	0.21	0.22	0.24	-0.21	-0.19	-0.18	-0.20	-0.22	-0.24
			PCQM3	0.21	0.19	0.17	0.19	0.20	0.21	-0.19	-0.17	-0.16	-0.18	-0.19	-0.21
		*Published*	PCQM1	0.43	0.40	0.45	0.44	0.40	0.44	-0.42	-0.39	-0.44	-0.44	-0.40	-0.44
			PCQM2	0.33	0.31	0.33	0.34	0.29	0.32	-0.31	-0.30	-0.32	-0.33	-0.29	-0.32
			PCQM3	0.28	0.25	0.28	0.29	0.24	0.27	-0.27	-0.24	-0.27	-0.28	-0.24	-0.27

In plant populations with “regular” spatial pattern, the RRMSE and RBIAS (Table 5) values show differences among the *corrected* PCQM versions when the repulsion distance is >75 cm but the values show no differences when the repulsion distance is <75 cm. When plants show very strong regularity (repulsion distance >75 cm), the *published* PCQM shows better performance than *corrected* PCQM (Table 5). The RBIAS values with *corrected* PCQM1 become more positive (deviated from zero) with increasing repulsion distance, but this tendency is less strong in the *corrected* PCQM2 and PCQM3. When repulsion distance is small the *corrected* PCQM performs better (Table 5).

**Table 5:** The relative root mean square error (RRMSE) and relative bias (RBIAS) with “regular” spatial pattern having varying repulsion distances (RD) and a fixed true density of 3000 ha^-1^.

RD (m)	PCQM type		RRMSE						RBIAS					
			Sample points						Sample points					
			15	20	25	30	50	100	15	20	25	30	50	100
0.25	*Corrected*	PCQM1	0.14	0.12	0.11	0.10	0.07	0.06	0.02	0.02	0.02	0.02	0.02	0.02
		PCQM2	0.09	0.08	0.07	0.07	0.05	0.03	0.00	0.00	0.00	0.00	0.00	0.00
		PCQM3	0.08	0.06	0.06	0.05	0.04	0.03	0.00	0.00	-0.01	-0.01	0.00	0.00
	*Published*	PCQM1	0.25	0.24	0.24	0.24	0.24	0.24	-0.22	-0.22	-0.23	-0.23	-0.23	-0.23
		PCQM2	0.14	0.14	0.14	0.13	0.13	0.12	-0.12	-0.12	-0.12	-0.12	-0.12	-0.12
		PCQM3	0.11	0.10	0.10	0.10	0.09	0.09	-0.08	-0.08	-0.08	-0.08	-0.08	-0.08
0.50	*Corrected*	PCQM1	0.16	0.15	0.14	0.13	0.12	0.11	0.09	0.09	0.09	0.09	0.09	0.09
		PCQM2	0.10	0.09	0.08	0.08	0.06	0.05	0.04	0.04	0.04	0.04	0.04	0.04
		PCQM3	0.07	0.07	0.06	0.06	0.04	0.04	0.02	0.02	0.02	0.02	0.02	0.02
	*Published*	PCQM1	0.11	0.11	0.10	0.10	0.10	0.09	-0.08	-0.08	-0.08	-0.08	-0.09	-0.09
		PCQM2	0.27	0.23	0.24	0.23	0.23	0.25	0.27	0.23	0.24	0.23	0.23	0.25
		PCQM3	0.09	0.08	0.08	0.07	0.07	0.07	-0.06	-0.06	-0.06	-0.06	-0.06	-0.06
0.75	*Corrected*	PCQM1	0.25	0.25	0.24	0.24	0.23	0.22	0.21	0.21	0.21	0.21	0.22	0.21
		PCQM2	0.12	0.12	0.12	0.12	0.11	0.11	0.10	0.10	0.10	0.10	0.10	0.10
		PCQM3	0.09	0.08	0.08	0.08	0.07	0.07	0.06	0.06	0.06	0.06	0.06	0.06
	*Published*	PCQM1	0.14	0.12	0.12	0.11	0.10	0.10	-0.08	-0.08	-0.08	-0.08	-0.09	-0.09
		PCQM2	0.08	0.07	0.07	0.06	0.05	0.05	-0.03	-0.04	-0.03	-0.03	-0.04	-0.04
		PCQM3	0.06	0.06	0.05	0.05	0.04	0.03	-0.02	-0.02	-0.02	-0.02	-0.03	-0.03
1.0	*Corrected*	PCQM1	0.42	0.41	0.41	0.41	0.41	0.40	0.39	0.39	0.39	0.40	0.40	0.40
		PCQM2	0.18	0.17	0.17	0.17	0.17	0.17	0.16	0.16	0.16	0.16	0.17	0.17
		PCQM3	0.12	0.11	0.11	0.11	0.11	0.11	0.11	0.10	0.11	0.11	0.11	0.11
	*Published*	PCQM1	0.12	0.11	0.11	0.10	0.08	0.07	0.06	0.06	0.06	0.06	0.05	0.05
		PCQM2	0.07	0.06	0.05	0.05	0.04	0.03	0.02	0.02	0.03	0.02	0.02	0.02
		PCQM3	0.05	0.05	0.04	0.04	0.03	0.02	0.02	0.02	0.02	0.02	0.02	0.02

In the natural plant population with an aggregated pattern (Site 1 in Table 1), the RRMSE and RBIAS values (Table 6) show no differences among the *corrected* PCQM versions. The RBIAS values are very close to ‘zero’. However, in the natural plant population with a repulsion (Site 2 & 3 in Table 1) the RRMSE and RBIAS values for the *corrected* PCQM are also close to ‘zero’. In the natural plant populations the *corrected* estimators again provide better RRMSE and RBIAS values (closer to ‘zero’) than the *published* estimators.

**Table 6:** The relative root mean square error (RRMSE) and relative bias (RBIAS) with “natural forests” having different true densities.

True density (ha^–1^)	PCQM type		RRMSE						RBIAS					
			Sample points						Sample points					
			15	20	25	30	50	100	15	20	25	30	50	100
15450	*Corrected*	PCQM1	0.11	0.10	0.09	0.09	0.07	0.06	-0.05	-0.04	-0.04	-0.05	-0.05	-0.04
(Site 1)		PCQM2	0.07	0.07	0.06	0.06	0.05	0.04	-0.03	-0.03	-0.03	-0.03	-0.03	-0.03
		PCQM3	0.07	0.06	0.06	0.06	0.05	0.05	-0.05	-0.04	-0.04	-0.04	-0.05	-0.04
	*Published*	PCQM1	0.29	0.28	0.28	0.28	0.28	0.28	-0.28	-0.27	-0.28	-0.28	-0.28	-0.28
		PCQM2	0.16	0.15	0.16	0.15	0.16	0.15	-0.15	-0.15	-0.15	-0.15	-0.15	-0.15
		PCQM3	0.13	0.13	0.13	0.13	0.12	0.12	-0.12	-0.12	-0.12	-0.12	-0.12	-0.12
9650	*Corrected*	PCQM1	0.13	0.10	0.10	0.09	0.08	0.06	0.04	0.03	0.04	0.04	0.04	0.04
(Site 2)		PCQM2	0.08	0.07	0.06	0.06	0.04	0.03	0.01	0.01	0.01	0.01	0.01	0.01
		PCQM3	0.07	0.06	0.05	0.05	0.04	0.03	-0.01	-0.02	-0.01	-0.01	-0.01	-0.01
	*Published*	PCQM1	0.23	0.22	0.22	0.22	0.22	0.22	-0.21	-0.21	-0.22	-0.21	-0.22	-0.22
		PCQM2	0.12	0.13	0.13	0.12	0.12	0.12	-0.10	-0.11	-0.11	-0.11	-0.11	-0.12
		PCQM3	0.11	0.10	0.10	0.10	0.10	0.10	-0.09	-0.09	-0.09	-0.09	-0.09	-0.09
795	*Corrected*	PCQM1	0.12	0.11	0.10	0.07	0.06	0.05	0.02	0.01	0.02	0.02	0.02	0.02
(Site 3)		PCQM2	0.08	0.07	0.06	0.05	0.04	0.03	-0.02	-0.02	-0.01	-0.02	-0.02	-0.02
		PCQM3	0.07	0.06	0.06	0.05	0.05	0.04	-0.03	-0.03	-0.03	-0.04	-0.04	-0.04
	*Published*	PCQM1	0.24	0.24	0.24	0.24	0.24	0.24	-0.22	-0.23	-0.23	-0.23	-0.24	-0.23
		PCQM2	0.15	0.15	0.15	0.14	0.14	0.14	-0.13	-0.13	-0.14	-0.14	-0.14	-0.14
		PCQM3	0.12	0.12	0.12	0.12	0.12	0.12	-0.11	-0.11	-0.11	-0.11	-0.11	-0.11

The Wilcoxon rank-sum test (equivalent to the Mann-Whitney U test) based on plant density data estimated by PCQM equations in 1000 simulations for each scenario confirms significant differences (*P*< 0.01) between the *corrected* and *published* estimators of PCQM for all the plant assemblages examined. For most of the cases except for a ‘repulsion’ with a high repulsion distance (>75 cm), the non-parametric one-way analysis of variance (Kruskal-Wallis test) reveals no significant differences (*P*> 0.05) among the estimated densities resulting from the *corrected* estimators of PCQM1, PCQM2 and PCQM3 when more than 50 sample points are considered. However, for some cases (smaller sample size or repulsion) when the *corrected* PCQM versions shows a significant difference (*P*< 0.05), a post-hoc test shows no significant differences (*P*> 0.05) between the *corrected* versions of PCQM2 and PCQM3.

## Discussion

The PCQM approach is generally accurate in randomly distributed populations [1, 2, 16]. In this study, the *corrected* PCQM shows the best performance with ‘random’ pattern and a reasonable performance for other plant assemblages, such as ‘aggregated’ and ‘regular’ patterns. The estimated results in natural plant populations with a semi-aggregated pattern and a semi-regular pattern (Table 1) suggest the applicability of the *corrected* PCQM estimators in natural plant populations. Only when plants show very strong regularity (repulsion distance >75 cm), the *published* PCQM shows better performance than *corrected* PCQM (Table 5). The repulsion distance means the minimum distance of closest neighbours, e.g. a plantation with seedlings planted at >75 cm intervals in all directions.

**Fig 3:**
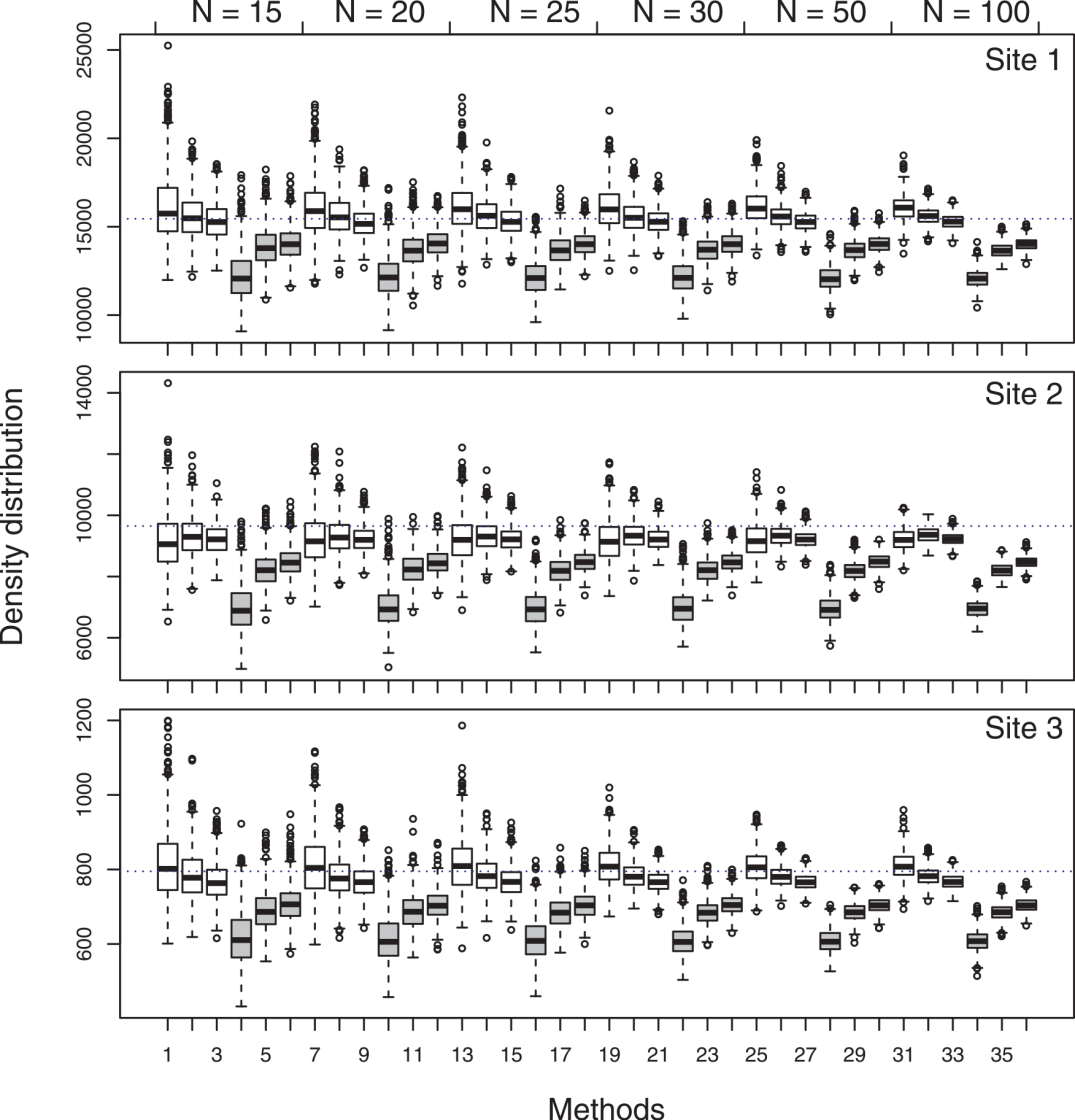
Box plot of the density (individuals ha^–1^) distribution of 1,000 simulations estimated with different methods using varying sample points (N = 15 to 100) comparing the differences between the two estimators in three natural populations (site 1, site 2 and site 3). In each sample size, boxes with white background represent *corrected* estimators and those with grey background represent *published* estimators (PCQM1, PCQM2 and PCQM3 from left to right in each scenario). The dotted horizontal line in each plot indicates the true density.

As presented in Table 3, we have used various “true” tree densities (2000, 5000, 10000 and 15000 trees ha^-1^) for random spatial pattern in order to explore the suitability of the PCQM method in different situations. The results suggest that the *corrected* PCQM yields consistently better results for various tree densities and that the density itself has little or no impact in the performance of the PCQM. Therefore, tree density was kept constant (3000 trees ha^-1^) for clustered and regular spatial patterns in order to focus on the effect of other variables, such as aggregation radius, aggregation intensity, repulsion distance, etc. The improved performance of *corrected* PCQM is also visible (Fig 3) for natural populations having different “true” tree densities (Table 6).

The ANOVA, root mean square error (RRMSE) and relative bias (RBIAS) are sensitive to outliers in the predicted values. However, the median values in the box plot (Fig 2), which are not sensitive to outliers, suggest no remarkable differences among the PCQM1, PCQM2 and PCQM3 in density prediction using the *corrected* estimators. This reveals that the first order PCQM offers accurate density estimations if the *corrected* estimators and more than 50 sample points are used, and if the spatial pattern is completely random. Across all patterns we found that our *corrected* estimators are more robust and the estimated densities closer to true density and therefore more accurate than *published* ones [3, 4]. It was also found that the *published* estimators always underestimate the density with respect to the true density when compared to the *corrected* estimators.

The Wilcoxon and Kruskal-Wallis tests used in this paper are based on sample sizes of the 1000 simulations for each scenario. We admit that a sample size of 1000 would show statistically significant differences even for non-biologically significant differences. The distribution of estimated density in 1000 simulations (Fig 3) in three natural populations suggests conspicuous improvement (S1 Table) in the *corrected* PCQM.

In this study, the obtained RRMSE values using the *published* estimators varied between 0.0 and 0.30 (Tables 3, 4, 5 and 6) which is in agreement with the reported RRMSE values for PCQM [3, 4]. This exemplifies the robustness of the NetLogo model codes [22] that we have used for implementing the PCQM techniques in a virtual environment. In addition, the estimated densities of natural populations were very close to the true values obtained in the field (revealed by the RBIAS values closeness to zero). This again validates our model, which mimics real populations in the field. Although there is little variation among PCQM1, PCQM2 and PCQM3 in the *corrected* estimators in empirical datasets, the results are very close to the true values, which is improvement with respect to the *published* estimators.

The results of this study suggest that the estimations of densities using the *corrected* estimators are more accurate than *published* ones in the cases of various plant spatial patterns examined. We confirm that the higher order PCQM (PCQM2 and PCQM3) shows better prediction of density but in most cases such as in random and aggregated spatial patterns, and in regular plant assemblages with a repulsion distance of <75 cm, the differences are not significant if the sample size (*N*) is greater than 50. However, the higher order PCQM shows significant differences (*P*< 0.05) among the *corrected* PCQM versions when the repulsion distance is >75 cm. Therefore, we reject the hypothesis that higher order PCQM offers higher accuracy in density prediction for plant assemblages with random or aggregated plants but we accept the hypothesis for plant assemblages having strong repulsion.

When using PCQM in the field, care should be taken to summarize the distance data based on ‘the inverse summation of squared distances’ but not ‘the summation of inverse squared distances’ as erroneously reported in [3] and [4] where PCQM1, PCQM2 and PCQM3 have been denoted as AO1Q, AO2Q and AO3Q, respectively. For each PCQM (PCQM1, PCQM2, PCQM3), only one plant must be measured from each quadrant (per sample point). In case of PCQM2, the distance from a sample point to only the 2nd nearest plant in each of four quadrants is measured. Therefore, the sampling time in field works for PCQM2 would be very close to that in PCQM1. The same is true for PCQM3, where the third-nearest plant in each quadrant are measured and the first-and-second-nearest plants are skipped.

## Conclusion

The *corrected* estimators (higher order PCQM) improve the accuracy of PCQM in comparison with *published* ones. Over 50 sample points, the accuracy of density estimations among PCQM1, PCQM2 and PCQM3 is not significantly different for most of the plant assemblages except for those with a strong repulsion (e.g. plantation). The PCQM3 offers, however, the best density estimations for all types of plant assemblages including the repulsion process. In practice, generally before starting vegetation survey, the spatial pattern of a plant association is unknown. Therefore, for field applications the use of PCQM3 along with the *corrected* estimator is recommended. However, for sparse plants the use of PCQM3 may pose practical limitations of field works, the use of PCQM2 or PCQM1 would be valid. Our *corrected* PCQM estimators improved density estimations in common plant assemblages. Future research should focus on the performance of the corrected PCQM estimators in comparison with other plot-less- and plot-based methods in various plant assemblages.

## Supporting Information

S1 Fig Field data on original tree positions in natural forests. Fig 1, Fig 2 and Fig 3 represent site 1, site 2 and site 3, respectively.
(PDF)

S1 File Raw data on results of 1000 simulations of tree density estimated by corrected and published PCQM equations using the NetLogo model with different true density (2000, 5000, 10000 and 15000 trees/ha) and “random” spatial pattern. First, second, third and fourth rows represent Tree-density, samples, PCQM-eqn and PCQM types, respectively.
(CSV)

S2 File Raw data on results of 1000 simulations of tree density estimated by corrected and published PCQM equations using the simulation model with “aggregated” spatial pattern. First, second, third, fourth and fifth rows represent cluster-radius, cluster-percent, samples, PCQM-eqn and PCQM types, respectively.
(CSV)

S3 File Raw data on results of 1000 simulations of tree density estimated by corrected and published PCQM equations using the simulation model with “regular” spatial pattern. First, second, third and fourth rows represent repulsion distance, samples, PCQM-eqn and PCQM types, respectively.
(CSV)

S4 File Raw data on results of 1000 simulations of tree density estimated by corrected and published PCQM equations using the simulation model with natural datasets (site 1, site 2 and site 3). First, second, third and fourth rows represent sites, samples, PCQM-eqn and PCQM types, respectively.
(CSV)

S1 Table Example datasets on PCQM1, PCQM2 and PCQM3 to prove the differences between the published and corrected versions of the PCQM equations through direct computation. Table 1, Table 2 and Table 3 represent example data on PCQM1, PCQM2 and PCQM3, respectively.
(PDF)

S1 Text NetLogo codes of model used for simulation of PCQM equations are provided. These codes can be used to generate PCQM sample points in a simulation area and plant density can be estimated using real field data coming from forests and simulated data in which plant distributions are generated to be random, aggregated or regular.
(PDF)
