# Supplementary material for: An Evaluation of the Plant Density Estimator the Point-Centred Quarter Method (PCQM) Using Monte Carlo Simulation
Source: PLoS One. 2016 Jun 23;11(6):e0157985. doi: 10.1371/journal.pone.0157985 (PMC4919016; doi:10.1371/journal.pone.0157985)

S1 Fig. Field data on original tree positions in natural forests. Fig 1, Fig 2 and Fig 3 represent site 1, site 2 and site 3, respectively.

An Evaluation of the Plant Density Estimator the Point-Centred Quarter Method (PCQM) Using Monte Carlo Simulation

Md Nabiul Islam Khan[*1,2,3], Renske Hijbeek[4,5], Uta Berger[2], Nico Koedam[4], Uwe Grueters[2], SM Zahirul Islam[3], Md Asadul Hasan[3], Farid Dahdouh-Guebas[1,4]

Table of contents

Contents Page

Fig 1. Field data on original tree positions in natural forest site 1 2

Fig 2. Field data on original tree positions in natural forest site 2 3

Fig 3. Field data on original tree positions in natural forest site 3 4

----

[1]Laboratory of Systems Ecology and Resource Management, Département de Biologie des Organismes, Faculté des Sciences, Université Libre de Bruxelles – ULB, Bruxelles, Belgium
[2]Institute of Forest Growth and Forest Computer Sciences, TU Dresden, Tharandt, Germany
[3]Forestry and Wood Technology Discipline, Khulna University, Khulna, Bangladesh
[4]Biodiversity and Ecology Research Unit, Faculty of Sciences and Bio-engineering Sciences, Vrije Universiteit Brussel – VUB, Brussels, Belgium
[5] Plant Production Systems, Wageningen University and Research Centre, , Wageningen, Netherlands

Fig 1. Field data on original tree positions in natural forest site 1

(Density = 15450 trees/ha)

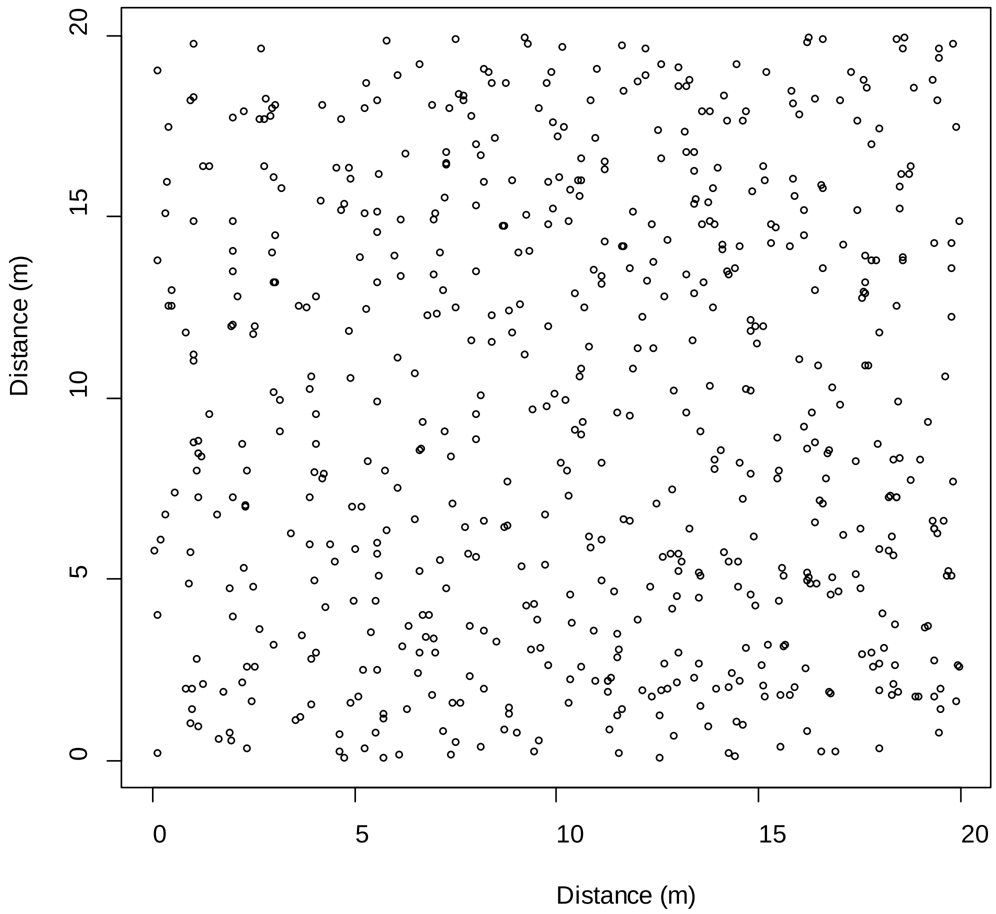

Fig 2. Field data on original tree positions in natural forest site 2

(density = 9650 trees/ha)

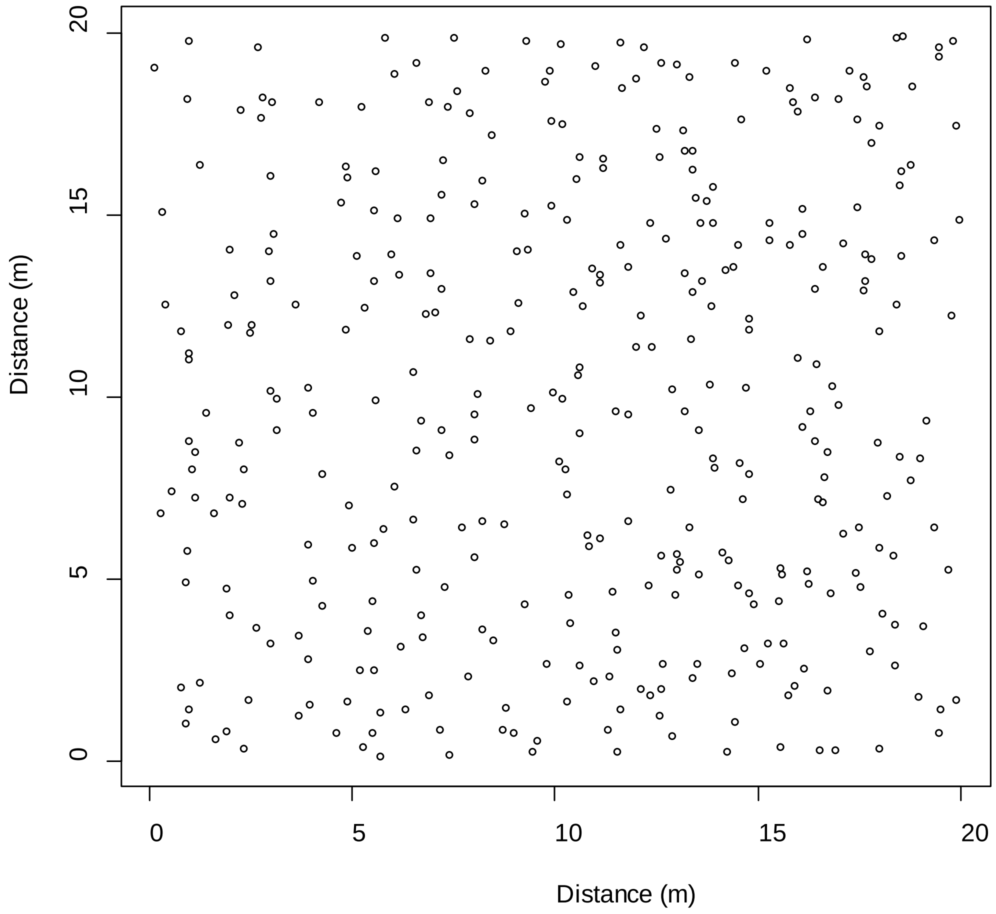

Fig 3. Field data on original tree positions in natural forest site 3

(density = 795 trees/ha)

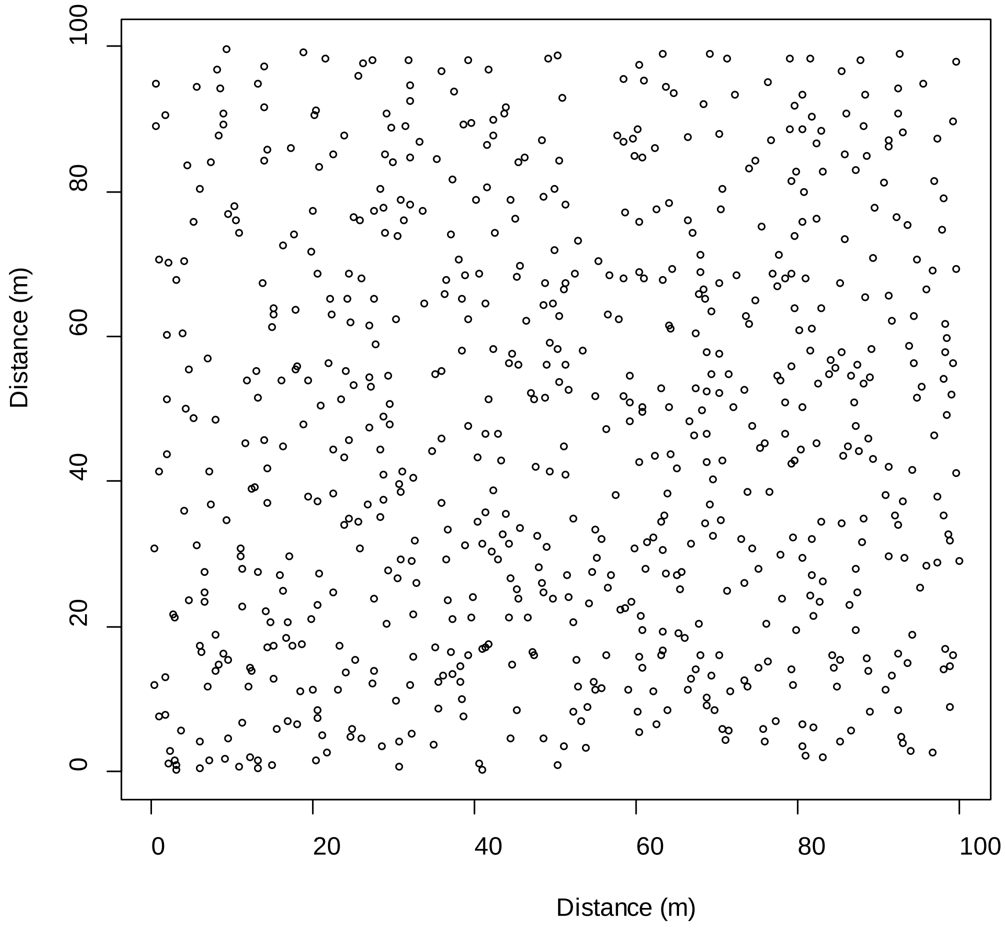

Supplement: S1 Fig — Fig 1, Fig 2 and Fig 3 represent site 1, site 2 and site 3, respectively. (PDF) [file pone.0157985.s001.pdf]
