# Supplementary material for: An Evaluation of the Plant Density Estimator the Point-Centred Quarter Method (PCQM) Using Monte Carlo Simulation
Source: PLoS One. 2016 Jun 23;11(6):e0157985. doi: 10.1371/journal.pone.0157985 (PMC4919016; doi:10.1371/journal.pone.0157985)
Supplement: S1 Table — Table 1, Table 2 and Table 3 represent example data on PCQM1, PCQM2 and PCQM3, respectively. (PDF) [file pone.0157985.s006.pdf]

S1 Table. Example datasets on PCQM1, PCQM2 and PCQM3 to prove the differences between the published and corrected versions of the PCQM equations through direct computation.

Table 1, Table 2 and Table 3 represent example data on PCQM1, PCQM2 and PCQM3, respectively.

An Evaluation of the Plant Density Estimator the Point-Centred Quarter Method (PCQM) Using Monte Carlo Simulation

Md Nabiul Islam Khan[*1,2,3], Renske Hijbeek[4,5], Uta Berger[2], Nico Koedam[4], Uwe Grueters[2], SM Zahirul Islam[3], Md Asadul Hasan[3], Farid Dahdouh-Guebas[1,4]

Table of contents

Contents Page

Table 1. Comparison of PCQM1 formulae with an example dataset from a randomly distributed population using "true density" of 1000 trees/ha and sample points *N* = 10................................ 2

Table 2. Comparison of PCQM2 formulae with an example dataset from a population using "true density" of 1000 trees/ha and sample points *N* = 10 3

Table 3. Comparison of PCQM3 formulae with an example dataset from a population using "true density" of 1000 trees/ha and sample points *N* = 10 4

----

[1]Laboratory of Systems Ecology and Resource Management, Département de Biologie des Organismes, Faculté des Sciences, Université Libre de Bruxelles – ULB, Bruxelles, Belgium
[2]Institute of Forest Growth and Forest Computer Sciences, TU Dresden, Tharandt, Germany
[3]Forestry and Wood Technology Discipline, Khulna University, Khulna, Bangladesh
[4]Biodiversity and Ecology Research Unit, Faculty of Sciences and Bio-engineering Sciences, Vrije Universiteit Brussel – VUB, Brussels, Belgium
[5] Plant Production Systems, Wageningen University and Research Centre, , Wageningen, Netherlands

Table 1. Comparison of PCQM1 formulae with an example dataset from a randomly distributed population using "TRUE density" of 1000 trees/ha and sample points *N* = 10.

	Sample	Distance (m)					Density estimated with **PCQM 1 (trees/ha)**	
	N	**d1**	**d2**	**d3**	**d4**	SUMSQ	**Published**	**Corrected**
**PCQM 1**	**1**	1.3	2.8	2.9	3.6			
	**2**	5.6	2	2.5	1.9			
	**3**	2.5	5.5	3.1	5.8			
	**4**	1.1	2.7	1.4	3.4			
	**5**	2.4	4.4	2.6	2.1			
	**6**	1.9	0.9	4.4	2.9			
	**7**	4.2	4.4	1.9	0.8			
	**8**	2	4.4	2	5.4			
	**9**	3.1	2.4	5	2.1			
	**10**	2	5	6.1	4.4	474.5	**805**	**1047**

$$SUMSQ = \sum (1.3^2 + 2.8^2 + \cdots + 4.4^2) = 474.5$$

$$PCQM1\ (published) = 10000 * \frac{12 * N}{\pi} * \frac{1}{SUMSQ} = 10000 * \frac{12 * 10}{\pi} * \frac{1}{474.5} = 805$$

$$PCQM1\ (corrected) = 10000 * \frac{4(4N - 1)}{\pi} * \frac{1}{SUMSQ} = 10000 * \frac{4(40 - 1)}{\pi} * \frac{1}{474.5} = 1047$$

[1 ha = 10,000 m^2]

Table 2. Comparison of PCQM2 formulae with an example dataset from a population using "TRUE density" of 1000 trees/ha and sample points N = 10

	**Sample**	Distance (m)					**Density estimated with PCQM 2 (trees/ha)**	
	N	d1	d2	d3	d4	SUMSQ	**Published**	**Corrected**
**PCQM 2**	**1**	3.1	3.6	5.4	4.4			
	**2**	6.2	2.2	2.7	2.1			
	**3**	5.5	7.2	4.2	5.8			
	**4**	4.8	3.5	1.7	6.2			
	**5**	3.6	4.7	2.7	4.2			
	**6**	2.3	5.9	6.1	4.3			
	**7**	5.5	5.3	2.9	2.6			
	**8**	3.6	5.9	5.3	7			
	**9**	5.6	6.5	5.6	7.7			
	**10**	2.9	6	8.1	5.8	998.1	**893**	**1008**

$$SUMSQ = \sum (3.1^2 + 3.6^2 + \cdots + 5.8^2) = 998.1$$

$$PCQM2\ (published) = 10000 * \frac{28 * N}{\pi} * \frac{1}{SUMSQ} = \qquad 10000 * \frac{28 * 10}{\pi} * \frac{1}{998.1} = 893$$

$$PCQM2\ (corrected) = 10000 * \frac{4(8N - 1)}{\pi} * \frac{1}{SUMSQ} = \qquad 10000 * \frac{4(80 - 1)}{\pi} * \frac{1}{998.1} = 1008$$

Table 3. Comparison of PCQM3 formulae with an example dataset from a population using "TRUE density" of 1000 trees/ha and sample points *N* = 10

	**Sample**	**Distance** (m)					Density estimated with **PCQM 3 (trees/ha)**	
	N	d1	d2	d3	d4	SUMSQ	**Published**	**Corrected**
**PCQM 3**	**1**	4.9	5.5	6.6	5.5			
	**2**	6.7	6.2	3.6	3.5			
	**3**	7.4	8.6	4.8	7			
	**4**	5.6	3.9	3.2	6.4			
	**5**	4.9	5.4	3.6	7.7			
	**6**	3	7	6.2	6.2			
	**7**	5.9	5.7	4.4	3.2			
	**8**	5.8	6.9	7.3	7.7			
	**9**	5.7	10.4	5.7	7.8			
	**10**	5.4	6.7	8.5	7.2	1518.3	**922**	**998**

$$SUMSQ = \sum (4.9^2 + 5.5^2 + \cdots + 7.2^2) = 1518.3$$

$$PCQM3\ (published) = 10000 * \frac{44 * N}{\pi} * \frac{1}{SUMSQ} = \quad 10000 * \frac{44 * 10}{\pi} * \frac{1}{1518.3} = 922$$

$$PCQM3\ (corrected) = \frac{4(12N - 1)}{\pi} * \frac{1}{SUMSQ} = \quad 10000 * \frac{4(120 - 1)}{\pi} * \frac{1}{1518.3} = 998$$
