# Supplementary material for: An Evaluation of the Plant Density Estimator the Point-Centred Quarter Method (PCQM) Using Monte Carlo Simulation
Source: PLoS One. 2016 Jun 23;11(6):e0157985. doi: 10.1371/journal.pone.0157985 (PMC4919016; doi:10.1371/journal.pone.0157985)
Supplement: S1 Text — These codes can be used to generate PCQM sample points in a simulation area and plant density can be estimated using real field data coming from forests and simulated data in which plant distributions are generated to be random, aggregated or regular. (PDF) [file pone.0157985.s007.pdf]

S1 Text. NetLogo codes of model used for simulation of PCQM equations are provided.

These codes can be used to generate PCQM sample points in a simulation area and plant density can be estimated using real field data coming from forests and simulated data in which plant distributions are generated to be random, aggregated or regular.

An Evaluation of the Plant Density Estimator the Point-Centred Quarter Method (PCQM) Using Monte Carlo Simulation

Md Nabiul Islam Khan[*1,2,3], Renske Hijbeek[4,5], Uta Berger[2], Nico Koedam[4], Uwe Grueters[2], SM Zahirul Islam[3] , Md Asadul Hasan[3], Farid Dahdouh-Guebas[1,4]

Table of contents

	Contents	Page
1.1	Declaration of global variables	2
1.2	Setup model	3
1.3	Start PCQM process	9
1.4	Simulate one Run	16
2.1	Export distance of PCQM sample trees as CSV file	18
2.2	Export X and Y position of trees as CSV file	20

----

[1]Laboratory of Systems Ecology and Resource Management, Département de Biologie des Organismes, Faculté des Sciences, Université Libre de Bruxelles – ULB, Bruxelles, Belgium

[2]Institute of Forest Growth and Forest Computer Sciences, TU Dresden, Tharandt, Germany

[3]Forestry and Wood Technology Discipline, Khulna University, Khulna, Bangladesh

[4]Biodiversity and Ecology Research Unit, Faculty of Sciences and Bio-engineering Sciences, Vrije Universiteit Brussel – VUB, Brussels, Belgium

[5] Plant Production Systems, Wageningen University and Research Centre, , Wageningen, Netherlands

1.1 Declaration of global variables

```
globals [
 PCQM-density
 PCQMtwo-density
 PCQMthree-density
 plot-based-density

 PCQM-density-new
 PCQMtwo-density-new
 PCQMthree-density-new


 $x      ; random x position for points
 $y      ; random y position for points
 d-data1 ; list of sum of distance data PCQM1
 d-data2 ; list of sum of distance data PCQM2
 d-data3 ; list of sum of distance data PCQM3
 Sample_point ; Number of sample points
 raw-pcqm1d1   ; raw distance data from each point to sample tree
 raw-pcqm1d2
 raw-pcqm1d3
 raw-pcqm1d4

 raw-pcqm2d1
 raw-pcqm2d2
 raw-pcqm2d3
 raw-pcqm2d4

 raw-pcqm3d1
 raw-pcqm3d2
 raw-pcqm3d3
 raw-pcqm3d4   ; raw distance data from each point to sample tree

 plot-data    ; square plot sample
 ]
```

1.2 Setup model

```
to setup


 __clear-all-and-reset-ticks
 ask patches [set pcolor 67]

 if spatial-structure = "random"
 [
  create-turtles round(initial-trees * world-width ^ 2 / 10000) ; slider initial-trees, 200-15000/ha
  [
   setxy random-xcor random-ycor
   set shape "circle"
   set color blue
   set size 0.65;random-normal 1 .2

  ]

  kill-additional-trees
 ]


  if spatial-structure = "repulsion"
 [
  let num round (initial-trees * world-width ^ 2 / 10000)

  while [num > 0]
  [
   let coordX random-xcor
   let coordY random-ycor
   let myNeighbors turtles with [distancexy coordX coordY <= repulsion-distance]
   let can_exist? true

   ask myNeighbors
   [
    if (distancexy coordX coordY) < repulsion-distance ; repulsion distance 0 to 1 m
    [ set can_exist? false   ]
   ]
   if (can_exist? = true)
```

```
    [ set num create_tree coordX coordY num ]
  ]

 kill-additional-trees
 ]



 if spatial-structure = "clustered"

[

     let trees-to-grow round ((initial-trees * world-width ^ 2 / 10000) * (1 - cluster-percent / 100) * 1.05)
     crt trees-to-grow  ; cluster-percent a slider 0-100
  [
  setxy random-xcor random-ycor
  set shape "circle"
  set color blue
  set size 0.65;random-normal 1 .2

  ]

  let num_cluster round(1.05 * (initial-trees * world-width ^ 2 / 10000) * (cluster-percent / 100) / 10) ; 10
offspring per cluster  ; White et al. 2008

  repeat num_cluster
  [
   let xx (random-float world-width * 0.95) - (world-width * 0.45)
   let yy (random-float world-height * 0.95) - (world-height * 0.45); (random-float 90) - 45

   crt 10 ;10 offspring per cluster  ; White et al. 2008
   [
     setxy xx yy set shape "circle"
     set color blue
     set size 0.65
     jump random-float cluster-radius      ; slider cluster-radius      as 0 to 5 m



   ]]

  kill-additional-trees
  ]
```

```
if spatial-structure = "uniform"

 [

   ;let rows   (0.5 * world-width / spacing-uniform-structure) ;* 10201 / 10000
   let rows round (sqrt (initial-trees * world-width ^ 2 / 10000))
   let spacing (world-width / rows)

   let mylist [];sentence n-values rows [? * spacing] n-values rows [? * -1 * spacing]
   let i 0
   let item-mylist min-pxcor ;+ 0.5

   while [i < rows]
   [
   set mylist lput item-mylist mylist
   set i i + 1
   set item-mylist (item-mylist + spacing)
   ]
   ;print mylist

   foreach mylist [
     let x1 ?
     foreach mylist [
       let y1 ?
       crt 1 [
         set xcor x1
         set ycor y1
         set size 0.65
         set color blue
         set shape "circle"]
     ]]

 ]



 if spatial-structure = "uniform-hexagonal"
```

```
  [

;let rows   (0.5 * world-width / spacing-uniform-structure) ;* 10201 / 10000
let rows round (sqrt (initial-trees * world-width ^ 2 / 10000)) * 0.7099976
let spacing (world-width - 1) / rows

let mylist [];sentence n-values rows [? * spacing] n-values rows [? * -1 * spacing]
let i 0
let item-mylist min-pxcor ;+ 0.5

while [i < rows]
[
set mylist lput item-mylist mylist
set i i + 1
set item-mylist (item-mylist + spacing)
]

foreach mylist [
 let x1 ?
 foreach mylist [
  let y1 ?
  crt 1 [
   set xcor x1
   set ycor y1
   set size 0.65
   set color blue
   set shape "circle"]
 ]]

; hexagonal trees

let mylist2 []
let item-mylist2 min-pxcor  - (item 0 mylist - item 1 mylist) / 2

let j 0
 while [j < (rows - 1)]
[
set mylist2 lput item-mylist2 mylist2
set j j + 1
set item-mylist2 (item-mylist2 + spacing)
]
```

```
        ;set mylist2 butfirst mylist2
;        print mylist
;        print mylist2


        foreach mylist2 [
         let x1 ?
         foreach mylist2 [
          let y1 ?
          crt 1 [
           set xcor x1
           set ycor y1
           set size 0.65
           set color blue
           set shape "circle"]
         ]]




    ]




if spatial-structure = "field-xy-positions"

    [

 let x-list (list )                    ;; This makes a list for storing the x-coordinates from the user file.
 let y-list (list )                    ;; This makes a list for storing the y-coordinates from the user file.
;  let diam-list (list )                    ;; This makes a list for storing the diameters from the user file.

 file-open (word "XY_LNP.txt")          ;; This opens the file designated by the user in the FILE-NAME prompt.
                                        ;; The file is a TAB DELIMITED TXT having TWO columns (x,y) without headings


 while [not file-at-end?][                 ;; This reads the file from the beginning to the end.
  set x-list fput file-read x-list          ;; This puts the x-coordinates in the x-list (file must be: x, y, diam).
  set y-list fput file-read y-list          ;; This puts the y-coordinates in the y-list.
;   set diam-list fput file-read diam-list     ;; This puts the diameter values in the diam-list.
 ]
```

```
 file-close                              ;; This closes the file designated by the user in the FILE-NAME prompt.



 (foreach x-list y-list  [                                    ;; This code loops through the x-, y-, and diam- lists to

    crt 1
    [                                              ;; create a tree with each set of x, y, and diameter values.
                        ;; place some trees on the world edge. The "add" calculations
      set xcor ?1                                  ;; scale the population inwards to prevent edge trees.
      set ycor ?2
      set color blue
      set shape "circle"                               ;; SETUP-TREE-VALUES sets up the remaining tree values.
;       set diameter ?3
      set size 0.65; diameter / 5
    ]
 ])
    ]
end

to kill-additional-trees    ; this is needed for creating clustered pattern to retain same population density

 let aa count turtles
 let bb round (initial-trees * world-width ^ 2 / 10000) ; trees kept in the world
 let trees-to-kill (aa - bb)
if trees-to-kill > 0[
 ask n-of trees-to-kill turtles [die]

]
end

to-report create_tree [ coordX coordY num]
 ; create just a dummy tree

 crt 1
 [
 setxy coordX coordY
 set size 0.65
 set color blue
 set shape "circle"
 ]
```

```
 set num num - 1

 report num

end
```

1.3 Start PCQM process

```
to do-pcqm ;[ pcqm_position ]   ;  pcqm_position (0,1,2)

 let $ne [] ; turtle list in 4 quadrants
 let $se []
 let $sw []
 let $nw []

 ; turtle counts in 4 quadrants
 let ne-turtles count turtles with [xcor > $x and ycor > $y]
 let se-turtles count  turtles with [xcor > $x and ycor < $y]
 let sw-turtles count turtles with [xcor < $x and ycor < $y]
 let nw-turtles count  turtles with [xcor < $x and ycor > $y]

 ;ask patches with [pxcor = round $x or pycor = round $y]  [set pcolor red ]


 ; find closest turtle to point in each quadrant
 if ne-turtles <= 1
 [set $ne min-one-of turtles with [xcor > $x and ycor > $y] [distancexy $x $y]]
     if ne-turtles = 2
     [ set $ne min-n-of 2 turtles with [xcor > $x and ycor > $y] [distancexy $x $y]]
        if ne-turtles >= 3
        [ set $ne min-n-of 3 turtles with [xcor > $x and ycor > $y] [distancexy $x $y]]

 if se-turtles <= 1
   [set $se min-one-of  turtles with [xcor > $x and ycor < $y] [distancexy $x $y]]
   if se-turtles = 2
```

```
    [set $se min-n-of 2  turles with [xcor > $x and ycor < $y] [distancexy $x $y]]
    if se-turtles >= 3
     [set $se min-n-of 3  turtles with [xcor > $x and ycor < $y] [distancexy $x $y]]

 if sw-turtles <= 1
 [set $sw min-one-of turtles with [xcor < $x and ycor < $y] [distancexy $x $y]]
 if sw-turtles = 2
 [set $sw min-n-of 2  turtles with [xcor < $x and ycor < $y] [distancexy $x $y]]
   if sw-turtles >= 3
   [set $sw min-n-of 3  turtles with [xcor < $x and ycor < $y] [distancexy $x $y]]

 if nw-turtles <= 1
 [set $nw min-one-of turtles with [xcor < $x and ycor > $y] [distancexy $x $y]]
 if nw-turtles = 2
 [set $nw min-n-of 2  turtles with [xcor < $x and ycor > $y] [distancexy $x $y]]
 if nw-turtles >= 3
   [set $nw min-n-of 3  turtles with [xcor < $x and ycor > $y] [distancexy $x $y]]




  ; create a PCQM mark with lines to seleted trees on the simulation area
 ask (turtle-set $ne $se $sw $nw) [ hatch 1 [set color yellow pd set pen-size 2 setxy $x $y die] ]

 ;ask turtles with [xcor = $x and ycor = $y] [die]  ; kill the dummy hatchlings



  let ne1  [] ; blank list for holding distance data
  let se1  []
  let sw1  []
  let nw1  []

  set ne1  map [[distancexy $x $y] of ?] filter [? != nobody] (list $ne )
  set se1  map [[distancexy $x $y] of ?] filter [? != nobody] (list $se )
  set sw1  map [[distancexy $x $y] of ?] filter [? != nobody] (list $sw )
  set nw1  map [[distancexy $x $y] of ?] filter [? != nobody] (list $nw )

let ne2 [] ; blank list for creating new list with same length, always 3 data including 0
let se2 []
let sw2 []
let nw2 []
```

```
if length ne1 = 0 [set ne2 [0 0 0 ]]
if length se1 = 0 [set se2 [0 0 0 ]]
if length sw1 = 0 [set sw2 [0 0 0 ]]
if length nw1 = 0 [set nw2 [0 0 0 ]]

if ne-turtles = 1 [set ne2 sentence ne1 list 0 0 ]  ; list is stored like [ data1 ]
if se-turtles = 1 [set se2 sentence se1 list 0 0 ]
if sw-turtles = 1 [set sw2 sentence sw1 list 0 0  ]
if nw-turtles = 1 [set nw2 sentence nw1 list 0 0 ]

if ne-turtles = 2 [set ne2 first ne1 set ne2 sentence  ne2 0] ; list is stored like [ [ data1 data2 ] ]
if se-turtles = 2 [set se2 first se1 set se2 sentence  se2 0]
if sw-turtles = 2 [set sw2 first sw1 set sw2 sentence  sw2 0]
if nw-turtles = 2 [set nw2 first nw1 set nw2 sentence  nw2 0]

if ne-turtles >= 3 [set ne2 first ne1 ]   ; list is stored like [ [ data1 data2 data3 ] ]
if se-turtles >= 3 [set se2 first se1 ]
if sw-turtles >= 3 [set sw2 first sw1 ]
if nw-turtles >= 3 [set nw2 first nw1 ]

let ne-pcqm-one 0 ; data for distance based on pcqm 1
let se-pcqm-one 0
let sw-pcqm-one 0
let nw-pcqm-one 0



; PCQM 1 data stored into temporary memory

 ifelse sum ne2 > 0
 [
   set ne-pcqm-one sort-by [?1 < ?2] ne2
   set ne-pcqm-one item 0 ne-pcqm-one   ; item 0 = pcqm1
   set ne-pcqm-one ( ne-pcqm-one ) ^ 2
 ]
 [ set ne-pcqm-one item 0 ne2] ; if there is no tree set to ZERO; sorting [0 0 0] not possible

 ifelse sum se2 > 0
 [
   set se-pcqm-one sort-by [?1 < ?2] se2
   set se-pcqm-one item 0 se-pcqm-one
   set se-pcqm-one ( se-pcqm-one ) ^ 2
```

```
 ]
 [ set se-pcqm-one item 0 se2] ; if there is no tree set to ZERO; sorting [0 0 0] not possible

 ifelse sum sw2 > 0
 [
   set sw-pcqm-one sort-by [?1 < ?2] sw2
   set sw-pcqm-one item 0 sw-pcqm-one
   set sw-pcqm-one ( sw-pcqm-one ) ^ 2
 ]
 [ set sw-pcqm-one item 0 sw2] ; if there is no tree set to ZERO; sorting [0 0 0] not possible

 ifelse sum nw2 > 0
 [
   set nw-pcqm-one sort-by [?1 < ?2] nw2
   set nw-pcqm-one item 0 nw-pcqm-one
   set nw-pcqm-one ( nw-pcqm-one ) ^ 2
 ]
 [ set nw-pcqm-one item 0 nw2] ; if there is no tree set to ZERO; sorting [0 0 0] not possible


let ne-pcqm-two 0 ; data for distance based on pcqm 2
let se-pcqm-two 0
let sw-pcqm-two 0
let nw-pcqm-two 0



; PCQM 2 data stored into temporary memory

 ifelse sum ne2 > 0
 [
   set ne-pcqm-two sort-by [?1 < ?2] ne2
   set ne-pcqm-two item 1 ne-pcqm-two   ; item 0 = pcqm1
   set ne-pcqm-two ( ne-pcqm-two ) ^ 2
 ]
 [ set ne-pcqm-two item 1 ne2] ; if there is no tree set to ZERO; sorting [0 0 0] not possible

 ifelse sum se2 > 0
 [
   set se-pcqm-two sort-by [?1 < ?2] se2
   set se-pcqm-two item 1 se-pcqm-two
```

```
    set se-pcqm-two ( se-pcqm-two ) ^ 2
  ]
  [ set se-pcqm-two item 1 se2] ; if there is no tree set to ZERO; sorting [0 0 0] not possible

  ifelse sum sw2 > 0
  [
    set sw-pcqm-two sort-by [?1 < ?2] sw2
    set sw-pcqm-two item 1 sw-pcqm-two
    set sw-pcqm-two ( sw-pcqm-two ) ^ 2
  ]
  [ set sw-pcqm-two item 1 sw2] ; if there is no tree set to ZERO; sorting [0 0 0] not possible

  ifelse sum nw2 > 0
  [
    set nw-pcqm-two sort-by [?1 < ?2] nw2
    set nw-pcqm-two item 1 nw-pcqm-two
    set nw-pcqm-two ( nw-pcqm-two ) ^ 2
  ]
  [ set nw-pcqm-two item 1 nw2] ; if there is no tree set to ZERO; sorting [0 0 0] not possible



let ne-pcqm-three 0 ; data for distance based on pcqm 3
let se-pcqm-three 0
let sw-pcqm-three 0
let nw-pcqm-three 0

; PCQM 3 data stored into temporary memory

  ifelse sum ne2 > 0
  [
    set ne-pcqm-three sort-by [?1 < ?2] ne2
    set ne-pcqm-three item 2 ne-pcqm-three   ; item 0 = pcqm1
    set ne-pcqm-three ( ne-pcqm-three ) ^ 2
  ]
  [ set ne-pcqm-three item 2 ne2] ; if there is no tree set to ZERO; sorting [0 0 0] not possible

  ifelse sum se2 > 0
  [
    set se-pcqm-three sort-by [?1 < ?2] se2
    set se-pcqm-three item 2 se-pcqm-three
    set se-pcqm-three ( se-pcqm-three ) ^ 2
```

```
 ]
 [ set se-pcqm-three item 2 se2] ; if there is no tree set to ZERO; sorting [0 0 0] not possible

 ifelse sum sw2 > 0
 [
   set sw-pcqm-three sort-by [?1 < ?2] sw2
   set sw-pcqm-three item 2 sw-pcqm-three
   set sw-pcqm-three ( sw-pcqm-three ) ^ 2
 ]
 [ set sw-pcqm-three item 2 sw2] ; if there is no tree set to ZERO; sorting [0 0 0] not possible

 ifelse sum nw2 > 0
 [
   set nw-pcqm-three sort-by [?1 < ?2] nw2
   set nw-pcqm-three item 2 nw-pcqm-three
   set nw-pcqm-three ( nw-pcqm-three ) ^ 2
 ]
 [ set nw-pcqm-three item 2 nw2] ; if there is no tree set to ZERO; sorting [0 0 0] not possible



let PCQM-density-sum1 ( ne-pcqm-one + se-pcqm-one + sw-pcqm-one + nw-pcqm-one )
let PCQM-density-sum2 ( ne-pcqm-two + se-pcqm-two + sw-pcqm-two + nw-pcqm-two)
let PCQM-density-sum3 ( ne-pcqm-three + se-pcqm-three + sw-pcqm-three + nw-pcqm-three )

    ; add multiple sample data into list
set d-data1 lput PCQM-density-sum1 d-data1
set d-data2 lput PCQM-density-sum2 d-data2
set d-data3 lput PCQM-density-sum3 d-data3



;;;   create distance data
let d1 sqrt ne-pcqm-one
let d2 sqrt se-pcqm-one
let d3 sqrt sw-pcqm-one
let d4 sqrt nw-pcqm-one

let d5 sqrt ne-pcqm-two
let d6 sqrt se-pcqm-two
let d7 sqrt sw-pcqm-two
let d8 sqrt nw-pcqm-two
```

```
let d9 sqrt ne-pcqm-three
let d10 sqrt se-pcqm-three
let d11 sqrt sw-pcqm-three
let d12 sqrt nw-pcqm-three

set raw-pcqm1d1 lput d1 raw-pcqm1d1
set raw-pcqm1d2 lput d2 raw-pcqm1d2
set raw-pcqm1d3 lput d3 raw-pcqm1d3
set raw-pcqm1d4 lput d4 raw-pcqm1d4

set raw-pcqm2d1 lput d5 raw-pcqm2d1
set raw-pcqm2d2 lput d6 raw-pcqm2d2
set raw-pcqm2d3 lput d7 raw-pcqm2d3
set raw-pcqm2d4 lput d8 raw-pcqm2d4

set raw-pcqm3d1 lput d9 raw-pcqm3d1
set raw-pcqm3d2 lput d10 raw-pcqm3d2
set raw-pcqm3d3 lput d11 raw-pcqm3d3
set raw-pcqm3d4 lput d12 raw-pcqm3d4


end



to do-plot-based-density ; create a 5x5 or 10x10 square plots around PCQM point for a reference


  ; create a red mark on the sample plot
  ;ask patches with [pxcor > round $x and pycor > round $y and pxcor <  round $x + plot-size and pycor < round $y + plot-size]  [set pcolor red ]
    ask patches with [pxcor > round ($x - plot-size * 0.5) and pycor > round ($y - plot-size * 0.5) and pxcor <  round $x + plot-size * 0.5 and pycor < round $y + plot-size * 0.5]  [set pcolor red ]

ifelse count turtles  < 2 [report 0 ]
[
  set plot-data lput (10000 * (1 / plot-size ^ 2) * count turtles with [ xcor > $x and ycor > $y and xcor <  $x + plot-size and ycor < $y + plot-size ]) plot-data   ;;; store density / ha
]

end
```

1.4 Simulate one Run

```
to simulate-one-replication ;[ pcqm_position ]

 set d-data1 []
 set d-data2 []
 set d-data3 []

 set raw-pcqm1d1 []
 set raw-pcqm1d2 []
 set raw-pcqm1d3 []
 set raw-pcqm1d4 []

 set raw-pcqm2d1 []
 set raw-pcqm2d2 []
 set raw-pcqm2d3 []
 set raw-pcqm2d4 []

 set raw-pcqm3d1 []
 set raw-pcqm3d2 []
 set raw-pcqm3d3 []
 set raw-pcqm3d4 []

 set Sample_point []



 set plot-data []

; #### create list for sample pints
  set Sample_point []; convert global value to list

  let j 1

      while [j <= samples]
      [
      set Sample_point lput j Sample_point
      set j j + 1
      ]
```

```
 let x-list []  ; local list to keep $x $y data
 let y-list []


   repeat samples
  [  ;  start position of loop

     set $x random-float (world-width * 0.8) - (world-width * 0.4)
     set $y random-float (world-width * 0.8) - (world-width * 0.4)
;     (A boundary strip of 10% of the length and width)


do-plot-based-density

do-pcqm


];;;;; end loop for plot


 let PCQM-density-sum1 sum d-data1
 let PCQM-density-sum2 sum d-data2
 let PCQM-density-sum3 sum d-data3


 ifelse PCQM-density-sum1 > 0
 [set PCQM-density  ( 10000 * 12 * samples * ( 1 / pi) * (1 / PCQM-density-sum1 ) ) ] [ set PCQM-density 0 ]

   ifelse PCQM-density-sum2 > 0
   [set PCQMtwo-density ( 10000 * 28 * samples * (1 / pi) * ( 1 / PCQM-density-sum2  ) ) ] [ set PCQMtwo-density
0 ]

       ifelse PCQM-density-sum3 > 0
       [set PCQMthree-density ( 10000 * 44 * samples * (1 / pi) * ( 1 / PCQM-density-sum3  ) ) ] [ set PCQMthree-
density 0 ]



 ifelse PCQM-density-sum1 > 0
 [set PCQM-density-new ( 10000 * (4 * (4 * samples - 1)) * (1 / pi) * ( 1 / PCQM-density-sum1  ) )] [ set PCQM-
density-new 0 ]
```

```
    ifelse PCQM-density-sum2 > 0
    [set PCQMtwo-density-new ( 10000 * (4 * (8 * samples - 1)) * (1 / pi) * ( 1 / PCQM-density-sum2  ) )] [ set
PCQMtwo-density-new 0 ]

        ifelse PCQM-density-sum3 > 0
        [set PCQMthree-density-new ( 10000 * (4 * (12 * samples - 1)) * (1 / pi) * ( 1 / PCQM-density-sum3  ) )] [ set
PCQMthree-density-new 0 ]

set plot-based-density mean plot-data

end
```

2.1 Export distance of PCQM sample trees as CSV file

```
Export distance of SAMPLE trees as CSV file
;;=================================================================

to save-distance

  user-message "Click OK to generate a CSV file saved on the same folder where this NetLogo file is located. Each
click generates a NEW file name. To STOP the process click HALT"

  file-close-all

let filename (word (substring(remove ":" date-and-time) 0 25)"PCQM distance data.csv")        ;; export distance
data in csv file
carefully [file-delete filename][]
file-open filename

 file-print ( word "Sample" "," "d1" "," "d2" "," "d3" "," "d4" )                                        ;; create header
of data output

 (
```

```
  foreach
 Sample_point
 raw-pcqm1d1
 raw-pcqm1d2
 raw-pcqm1d3
 raw-pcqm1d4

 [ file-print ( word ?1 "," ?2 "," ?3 "," ?4 "," ?5) ]
 )


 (
  foreach
 Sample_point
 raw-pcqm2d1
 raw-pcqm2d2
 raw-pcqm2d3
 raw-pcqm2d4

 [ file-print ( word ?1 "," ?2 "," ?3 "," ?4 "," ?5) ]
 )


 (
  foreach
 Sample_point
 raw-pcqm3d1
 raw-pcqm3d2
 raw-pcqm3d3
 raw-pcqm3d4

 [ file-print ( word ?1 "," ?2 "," ?3 "," ?4 "," ?5) ]
 )

file-close-all

end
```

2.2 Export X and Y position of trees as CSV file

```
;; export X and Y position of trees as CSV file
to create-tree-map

 user-message "Click OK to generate a CSV file saved on the same folder where this NetLogo file is located. Each click generates a NEW file name. To STOP the process click HALT"

 file-close-all

let filename (word (substring(remove ":" date-and-time) 0 25)" Export XY of trees.csv")        ;; export X and Y position in csv file
carefully [file-delete filename][]
file-open filename

 file-print ( word "x" "," "y"  )                                        ;; create header of data output

 ask turtles
 [
 let mylist1 []
 let mylist2 []

 set mylist1 lput [ xcor ] of self mylist1
 set mylist2 lput [ ycor ] of self mylist2

 (foreach
   mylist1
   mylist2

   [ file-print ( word ?1 "," ?2) ])

 ]

 file-close-all

end
```

---------------- END -----------------------
